# Self-Assembled Cannabigerol-Based Nanoparticles: Design, Synthesis, and Antiproliferative Activity

**DOI:** 10.3390/pharmaceutics17050636

**Published:** 2025-05-11

**Authors:** Arianna Amenta, Giulia Nordio, Francesco Piazzola, Maria Luisa Di Paolo, Fabio Milani, Martina Giacomini, Andrea Citarella, Umberto Ciriello, Giuseppe Paladino, Sara Pellegrino, Federica Silvestri, Valerio Fasano, Lisa Dalla Via, Daniele Passarella

**Affiliations:** 1 Department of Chemistry, University of Milan, Via Golgi 19, 20133 Milano, Italy; arianna.amenta@unimi.it (A.A.); fabio.milani4@studenti.unimi.it (F.M.); martina.giacomini@studenti.unimi.it (M.G.); valerio.fasano@unimi.it (V.F.); daniele.passarella@unimi.it (D.P.); 2 Department of Pharmaceutical and Pharmacological Sciences, University of Padova, via F. Marzolo 5, 35131 Padova, Italy; giulia.nordio.1@studenti.unipd.it (G.N.); francesco.piazzola@unipd.it (F.P.); 3 Department of Molecular Medicine, University of Padova, via G. Colombo 3, 35131 Padova, Italy; marialuisa.dipaolo@unipd.it; 4 LINNEA SA, 6595 Riazzino, Switzerland; uciriello@linnea.ch (U.C.); gpaladino@linnea.ch (G.P.); 5 Department of Pharmaceutical Science, University of Milan, Via Golgi 19, 20133 Milano, Italy; sara.pellegrino@unimi.it; 6 Department of Industrial Engineering, University of Padova, Via Marzolo 9, 35131 Padova, Italy; federica.silvestri.3@phd.unipd.it

**Keywords:** cannabigerol, nanoparticles, cancer, drug delivery, targeted cancer therapy, synthesis, organic chemistry, medicinal chemistry

## Abstract

**Background/Objectives:** Cannabigerol (CBG) is a non-psychoactive phytocannabinoid with significant therapeutic potential, showing emerging applications in drug delivery. This study aimed to develop and evaluate CBG-conjugated nanoparticles (NPs) incorporating tubulin-targeting drugs to enhance anticancer activity. **Methods:** CBG was conjugated with *N*-desacetylthiocolchicine, paclitaxel, and camptothecin using sebacic acid and 4,4′-dithiodibutyric acid as linkers, and nanoparticles were obtained. The NPs were characterized by their stability and size (hydrodynamic diameters < 90 nm). Their antiproliferative activity was assessed in three human tumor cell lines and non-tumorigenic cells. Their cellular uptake and mechanisms of action were investigated via confocal microscopy and cell cycle analysis. **Results:** The chemical composition of the linkers significantly influenced the antiproliferative effect, with the NPs containing 4,4′-dithiodibutyric acid demonstrating higher activity. Notably, NP3b, formulated with this linker, exhibited up to an 80-fold increase in antiproliferative potency compared to its sebacic acid counterpart (NP3a). In mesothelioma cells (MSTO-211H), NP3b displayed significantly higher cytotoxicity than in non-tumorigenic mesothelial cells (MeT-5A), indicating selectivity for cancer cells. Further analysis in glioblastoma cells confirmed that the NPs retained the microtubule-disrupting effects of their parent drugs. **Conclusions:** These findings highlight the potential of CBG-based NPs as versatile nanomedicine platforms for targeted cancer therapy. This study underscores the importance of linker chemistry in modulating therapeutic efficacy and supports the development of multifunctional drug delivery systems.

## 1. Introduction

Lipid-based self-assembling nanoparticles (NPs) formed through the spontaneous aggregation of drug conjugate compounds in aqueous environments offer significant advantages in medicinal chemistry, particularly for improving drug bioavailability and targeted delivery [1]. These include high and adjustable drug-loading capacities, customizable physicochemical properties through molecular design, straightforward production processes, and improved biocompatibility due to the absence of cytotoxic and immunogenic risks associated with inert carriers [2,3]. Natural compounds offer a rich repository of molecules capable of inducing self-assembly, leading to the formation of nanoparticles with inherent therapeutic properties. These molecules, by virtue of their unique chemical structures, can spontaneously organize into nanoscale architectures, facilitating applications in drug delivery, imaging, and therapy [4]. Among these natural compounds, cannabinoids, which are bioactive constituents of the cannabis plant, have garnered significant attention [5]. Their amphiphilic nature enables them to self-assemble into nanostructures, enhancing their bioavailability and therapeutic efficacy. Research has demonstrated that cannabinoids can form stable nanoparticles, which may improve their solubility and targeted delivery in medical applications [6]. This self-assembly behavior not only optimizes the pharmacokinetic profiles of cannabinoids but also opens avenues for developing novel nanomedicine strategies. Cannabigerol (CBG), a non-psychoactive phytocannabinoid, has emerged as a promising candidate. CBG was first isolated in 1964 from a hexane extract of hashish, the resin of the female *Cannabis sativa* L. plant [7]. CBG is formed through the non-enzymatic decarboxylation of cannabigerolic acid and is typically present in low concentrations (<1%) in cannabis plants, as it readily converts into *trans*-Δ^9^-tetrahydrocannabinol (THC) and cannabidiol. CBG-rich plants have been cultivated through selective breeding, allowing for its extraction in significant quantities for industrial and pharmaceutical applications [7].

CBG has shown therapeutic potential in treating glaucoma and neurological disorders, primarily due to its ability to cross the blood–brain barrier. This characteristic allows CBG to reduce intraocular pressure and exert neuroprotective effects, which are beneficial in managing these conditions [8]. It exhibits low affinity for CB1 and CB2 receptors but acts as an agonist for both, demonstrating non-toxic properties and making it suitable for treating various diseases [9]. Recently, the integration of CBG into nanoparticles has been explored through the formulation of drug carrier nanoparticles using Pluronic-F127. These nanoparticles have demonstrated enhanced thermal stability, favorable drug-release profiles, and high entrapment efficiency. Furthermore, they were incorporated into 3D-printed sodium alginate films for potential wound healing applications [10].

Inspired by these advances, and building on our interest in exploring natural compounds for multifunctional applications [11,12,13,14,15,16], in the present work, we investigated CBG as both a self-assembly inducer and a therapeutic agent. Based on this dual functionality, we aimed to leverage the unique properties of CBG for the development of innovative nanomaterials with enhanced therapeutic potential. We conjugated CBG with tubulin-targeting drugs, including *N*-desacetylthiocolchicine (Desa), paclitaxel (PTX), and camptothecin (CPT), using sebacic acid and 4,4′-dithiodibutyric acid as linkers. The resulting conjugates formed stable NPs with hydrodynamic diameters of <90 nm, optimal for their use as drug delivery platforms. These NPs were evaluated for their antiproliferative effects on three human tumor cell lines (LN229, HT-29, and MSTO-211H) and on non-tumorigenic mesothelium cells (MeT-5A). Cell cycle and confocal microscopy analyses confirmed successful drug targeting in LN229, demonstrating the highest sensitivity to CBG treatment. This is of particular interest for microtubule-targeting agents because it was reported that the expression of β-III tubulin in glial tumors correlates with malignancy, and the downregulation of β-III tubulin inhibits the migration and invasion of glioblastoma cells [17]. This study highlights CBG’s versatility as both a self-assembly driver and therapeutic agent, paving the way for its application in next-generation nanomedicine.

## 2. Materials and Methods

### 2.1. Chemistry

**Figure 1:**
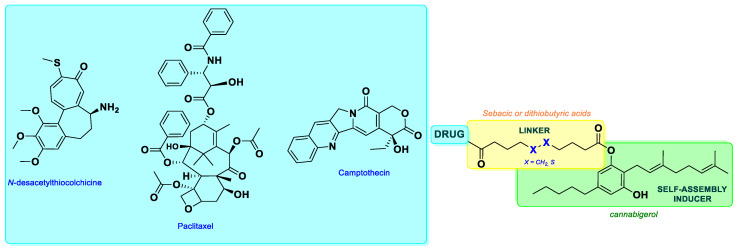
Design of cannabigerol-based drug conjugates.

Cannabigerol was provided by LINNEA SA. NMR spectra were recorded on a Bruker Advance instrument. ^1^H NMR spectra were recorded at 400 MHz, and ^13^C NMR spectra were recorded at 100 MHz, VTU 298.0 K. Chemical shifts were reported as parts per million (ppm) relative to the internal standard Me_4_Si. The ^1^H NMR spectra are described by reporting, for each signal, their chemical shift, multiplicity, integration, attribution (also using bi-dimensional experiments COSY and HSQC), and coupling constants. The ^13^C NMR spectra are described by reporting, for each signal, their chemical shift. The following abbreviations are used to describe ^1^H NMR spin multiplicity: s = singlet; d = doublet; t = triplet; q = quartet; m = multiplet; dd = doublet of doublets; td = double triplet; bs = broad singlet. HRMS data were analyzed on a Micromass Autospec LCT Premier XE orthogonal acceleration time-of-lap spectrometer (oa-TOF). For analytical thin-layer chromatography (TLC), silica gel pre-coated aluminum foils or glass plates were used. The plates were developed with 254 nm UV light and/or spraying with a solution of molybdic reagent (8.4 g of (NH_4_)_6_Mo_7_O_24_·4H_2_O, 1 g of Ce(SO_4_)_2_, 12.5 mL of 98% H_2_SO_4_, 187 mL of H_2_O) and heating, potassium permanganate stain (6 g of KMnO_4_, 40 g of K_2_CO_3_, 5 mL of 10% NaOH, 600 mL of H_2_O) and heating, or a solution of ninhydrin (0.2 g of ninhydrin, 100 mL of EtOH). Flash chromatography was carried out on SiO_2_ (Merck Grade, 60 Å pore size, 230–400 mesh particle size, Sigma Aldrich, St. Louis, MO, USA). All reagents were obtained from commercial sources, Sigma Aldrich, Fluorochem (Hadfield, Derbyshire, United Kingdom), and TCI (Tokyo, Japan), and used without any further purification. The reactions requiring anhydrous conditions were performed under a nitrogen atmosphere. All solvents were of reagent grade or HPLC grade. Dry THF, Py, CH_2_Cl_2_, and DMF were purchased from Sigma Aldrich. Figure 1 was created using ChemDraw Professional 17.0, which was also used for all the chemical structures depicted in our article (schemes).

### 2.2. Preparation and Characterization of Nanoparticles

Nanosuspensions were prepared at physiological pH in accordance with standard evaporation protocols. The drug conjugate was first dissolved in EtOH in a vial at room temperature. The resulting solution was added dropwise to a round-bottom flask containing MilliQ grade distilled water under stirring on a magnetic plate. The resulting suspension was stirred for additional 5 min, and then the EtOH was thoroughly evaporated under reduced pressure. The size, polydispersity index (PI), and ζ-potential were analyzed by dynamic light scattering (DLS) with a Zetasizer (Malvern Panalytical, Malvern, UK). Scanning electron microscopy (SEM) measurements were performed using a JEOL JSM-7900F High-Resolution Field Emission Gun Scanning Electrode Microscope, with a working distance of 10 mm and an applied acceleration voltage of 15 kV. SEM samples were prepared by dropping the nanoparticle dispersion on an aluminum cylindrical support and analyzed after drying overnight at room temperature.

### 2.3. Cell Cultures and Cell Viability Assay

The cell lines used in our study were purchased from ATCC (American Type Culture Collection). Specifically, we used LN-229 (ATCC CRL-2611), HT-29 (ATCC HTB-38), and MSTO-211H (ATCC CRL-2081). MeT-5A (ATCC CRL-9444), MSTO-211H (human biphasic mesothelioma), and Met-5A (human mesothelium) cells were grown in RPMI 1640 (R6504, Merck) supplemented with 2.38 g/L Hepes (H3375, Merck, St. Louis, MO, USA), 0.11 g/L pyruvate sodium (P5280, Merck), 2.5 g/L glucose (G6152, Merck), and 10% heat-inactivated fetal calf serum (FCS, F7524, Merck). HT-29 (human colorectal adenocarcinoma) cells were grown in RPMI 1640 (R6504, Merck) supplemented with 10% heat-inactivated FCS. LN229 (human glioblastoma) cells were cultured in DMEM (D2902, Merck) supplemented with 3.5 g/L glucose and 5% FCS. NaHCO_3_ (S5761, Merck), as indicated in the manufacturer’s instructions, 100 U/mL penicillin, 100 μg/mL streptomycin, and 0.25 μg/mL amphotericin B (A5955, Merck) were added to the culture media. The cells were maintained at 37 °C in a humidified atmosphere incubator containing 5% carbon dioxide in air.

The cell viability was analyzed by a trypan blue dye exclusion assay. Briefly, 3–4 × 10^4^ cells were seeded in each well of a 24-well cell culture plate and treated with test drugs or NPs at concentrations ranging from 1 to 30 nM and from 0.01 to 20 μM, respectively, after 24 of growth in standard conditions. After 72 h from the treatment, the cells were detached by trypsinization and counted by optical microscope. The data were expressed as GI_50_ values, that is, the concentration of test agent causing a 50% reduction in the cell number with respect to the untreated cells (control culture).

### 2.4. Confocal Microscopy

LN229 cells (3 × 10^4^) were seeded in 4-well cell culture chamber on a glass slide and cultured up to 50–60% confluence. The cells were then incubated for 4 h in the presence of Desa, **Np1b**, PTX, and **Np2b** at the indicated concentrations. After incubation, the cells were gently washed twice with PBS, fixed with 4% formaldehyde at room temperature for 15 min, washed twice with PBS, and permeabilized with 0.1% Triton X-100 (T8787, Merck) in PBS for 5 min in the dark. Again, the cells were washed twice with PBS and incubated for 30 min in the dark with 7% FCS in PBS. After the removal of FCS, the cells were stained with Alexa Fluor 488 mouse anti-β-tubulin (BD Pharmingen, San Diego, CA, USA) for 1 h at room temperature. After washing twice with PBS, 0.001 mg/mL DAPI (MBD0015, Merck) was added for 15 min, then the cells were washed with PBS, and the coverslips were mounted on glass slides using Fluoroshield^TM^ (F6182, Merck). Images were acquired through a Zeiss mod. LSM800 confocal microscope and the analysis was performed using Fiji (ImageJ, version 2.14.0), a distribution of ImageJ. Particle counting and size distribution were estimated using the built-in Analyze Particles plugin in Image J (Fiji).

### 2.5. Cell Cycle Analysis

A total of 2 × 10^5^ LN229 cells were seeded in Petri culture plates, left to grow for 24 h in standard conditions, and then treated with CPT and **Np3b** at the indicated concentrations. After 48 h, the cells were harvested, centrifuged, combined with ice-cold 70% *w*/*v* ethanol, and left at 4 °C for 20 min. Then, the cells were centrifuged, and the pellet was resuspended in a final volume of 300 μL PBS containing 0.1 mg mL^−1^ RNAse (R6513, Merck) and 42 mg mL^−1^ propidium iodide (P4170, Merck). The DNA content was quantified by a BD FACSAria III flow cytometer (BD Life Sciences) and the data were analyzed by BD FACSDiva software (version 9.0). Statistical analysis was performed by Student’s *t*-test.

## 3. Results and Discussion

### 3.1. Design

The main focus of this work was the synthesis of self-assembly drug conjugates using CBG as a self-assembly inducer (Figure 1). CBG possesses all the requisites to act as a self-assembly inducer, including biocompatibility, lipophilicity, and also the ability to cross the blood–brain barrier (BBB) [6]. Moreover, it has been reported that CBG presents many therapeutic properties, such as analgesic, anti-inflammatory, antioxidant, antimicrobial, anticancer, antidiabetic, and neuroprotective effects [7,18,19]. Therefore, we also considered the use of nanoparticles with a multiple or enhanced therapeutic response, due to the contemporary presence of CBG and the drug. *N*-desacetylthiocolchicine (Desa), paclitaxel (PTX), and camptothecin (CPT) were used as the active moieties, and sebacic acid and 4,4′-dithiodibutyrric acid were selected as the linkers (Figure 1). The linker 4,4′-dithiodibutyrric acid particularly captured our interest due to the presence of a disulfide bond, which could be selectively cleaved and reduced by glutathione (GSH), a tripeptide overexpressed in cancer cells, facilitating the release of the drug in the tumor microenvironment [20]. Finally, six drug conjugates (**Np1a**, **Np1b**, **Np2a**, **Np2b**, **Np3a**, and **Np3b**) bearing CBG as a self-assembly inducer were designed (Figure 1).

### 3.2. Synthesis

**Scheme 1:**
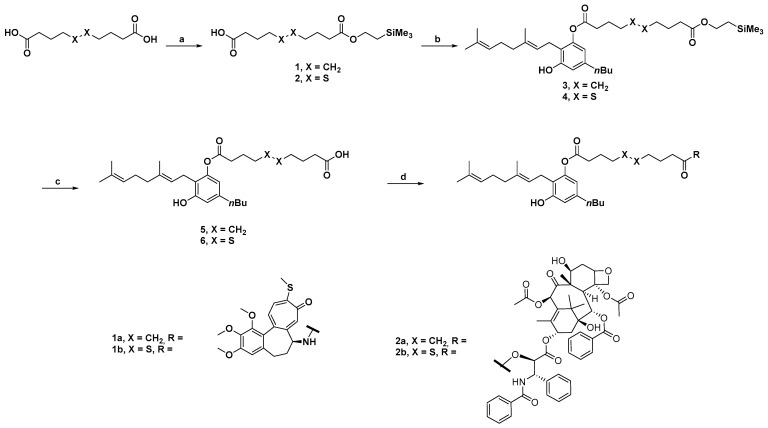
Synthesis of the final products **1a**, **1b**, **2a**, and **2b**. Reagents and conditions: (a) 2-(trimethylsilyl)ethanol, EDCI, DMAP, dry DCM/pyridine, rt, overnight, 66%; (b) CBG, EDCI, DMAP, dry DCM, rt, overnight, 43–57%; (c) TBAF, dry THF, rt, 4 h, 60–77%; (d) Desa (**1a** and **1b**) or paclitaxel (**2a** and **2b**), EDCI, DMAP, dry DCM/DMF, rt, overnight, 8–36%.

The synthesis of Desa and PTX conjugates **1a**, **1b**, **2a**, and **2b** is reported in Scheme 1. Sebacic acid or 4,4′-dithiodibutyrric acid were protected at a single carboxylic acid group by forming a TMSE ester using 2-(trimethylsilyl)ethanol in the presence of EDCI and DMAP. This reaction provided intermediates **1** (X = CH_2_) and **2** (X = S), both with a yield of 66%. Intermediates **2** and **3** were then reacted with CBG in an esterification reaction mediated by EDCI and DMAP, resulting in the formation of compounds **3** (X = CH_2_) and **4** (X = S), with moderate yields of 57% and 43%, respectively. The deprotection of **3** and **4** with TBAF in dry THF afforded free carboxylic acid intermediates **5** (X = CH_2_) and **6** (X = S), with yields of 77% and 60%, respectively. Finally, **5** and **6** were reacted with the appropriate Desa or PTX to afford the final products **1a**, **1b**, **2a**, and **2b**, with yields of 8–36% range.

**Scheme 2:**
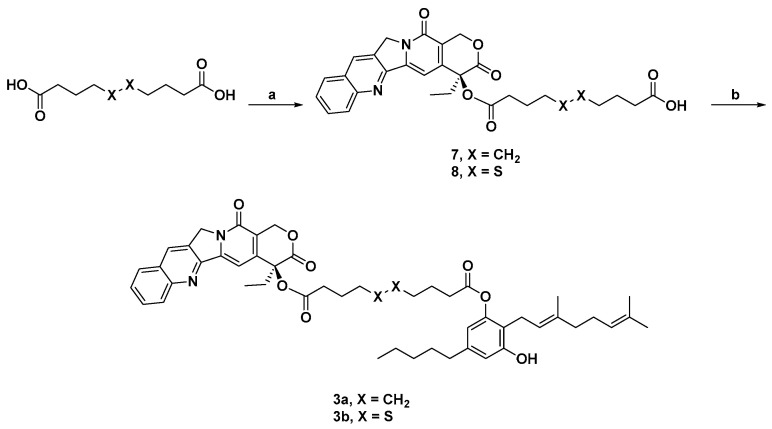
Synthesis of the final products **3a** and **3b**. Reagents and conditions: (a) CPT, EDCI, DMAP, dry DCM, rt, overnight, 56%, 64%; (b) CBG, EDCI, DMAP, dry DCM, rt, overnight, 16%, 51%.

The synthesis of CPT conjugates **3a** and **3b** is reported in Scheme 2. In this case, a different approach was pursued to obtain the final products. Sebacic acid or 4,4′-dithiodibutyrric acid were reacted with CPT in an esterification reaction in the presence of EDCI and DMAP, resulting in the formation of compounds **7** (X = CH_2_) and **8** (X = S) with moderate yields of 56% and 64%, respectively. The bicarboxylic acid starting material was used in excess (2.5 equiv) in order to avoid the formation of the bis-esterified side product. Finally, **7** and **8** were reacted with CBG to afford the final products **3a** and **3b**, with yields of 16 and 51%, respectively. The structural identity and the purity of all the synthesized compounds was assessed by ^1^H NMR, ^13^C NMR, and HRMS.

### 3.3. Nanoparticle Preparation and Characterization

To evaluate the self-assembly capabilities imparted by CBG to the synthesized conjugates, **1a**, **1b**, **2a**, **2b**, **3a**, and **3b**, nanoparticles (NPs) were prepared and characterized (Table 1). The preparation of the nanosuspensions followed standard solvent evaporation protocols [21], with one suspension produced for each drug conjugate at a concentration of 100 µM. Details of the preparation are reported in the Materials and Methods Section. The resulting NP suspensions were analyzed for their physicochemical properties, through dynamic light scattering (DLS), and the results are reported in Table 1.

**Figure 2:**
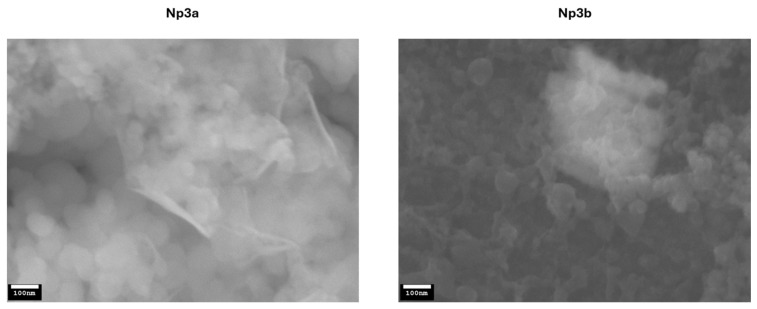
SEM images of **Np3a** and **Np3b** at 100,000x magnification.

All six CBG–drug conjugates formed monodisperse nanoparticles (PI < 0.2) with optimal hydrodynamic diameters ranging from 61 to 88 nm. Additionally, the negative zeta potential values observed for all nanoassemblies suggest that electrostatic repulsion plays a key role in ensuring the stability of the suspensions. To further characterize the morphology of the nanoparticles, SEM analysis was performed on representative formulations **Np3a** and **Np3b** (Figure 2). The images confirm the spherical shape and nanoscale dimensions of the particles, consistent with DLS data. Size distribution graphs have been included in the Supplementary Information (Figure S3).

### 3.4. Biological Evaluation

The NPs and the building blocks were assayed for their antiproliferative activity on three human tumor cell lines, LN229 (human glioblastoma), HT-29 (colorectal adenocarcinoma), and MSTO-211H (human biphasic mesothelioma), and on non-tumorigenic mesothelial cells, MeT-5A, after 72 h of incubation. The obtained results are shown in Table 2 as GI_50_ values, i.e., a 50% reduction in the viable cell number with respect to a control culture. The curves obtained by non-linear regression analysis of the compounds vs. the normalized response of some of the representative compounds are reported in Figure S1.

Our interest was turned to these cell lines because CBG demonstrated viability inhibition and apoptosis induction on glioblastoma cell lines [22] and a reduction in viability on colorectal carcinoma cell lines [23]. Moreover, a comparison between the antiproliferative effect on non-tumorigenic mesothelial cells (MeT-5A) derived from the mesothelium of healthy individuals and on human tumor cell line (MSTO-211H) derived from a patients with biphasic mesothelioma highlighted a possible specificity toward cancer vs. healthy cells.

The obtained results indicate, as expected, a strong antiproliferative effect by the reference cytotoxic compounds, Desa, PTX, and CPT, with GI_50_ values ranging from 0.0028 µM for PTX on HT-29 to 0.021 µM for Desa on LN229 cells. CBG also exerted a significant cytotoxic effect, although lower with respect to the above agents, with GI_50_ values in the low micromolar range (5.9–16 µM). This is in accordance with the literature data [22,24,25] and particularly confirms its effectiveness on glioblastoma LN229 cells, for which the lowest GI_50_ value (5.9 μM) was found. All these agents are devoid of any selectivity, and indeed, negligible differences were found between the mesothelioma (MSTO-211H) and mesothelial (MeT-5A) cells.

Concerning the NPs, they showed intermediate GI_50_ values with respect to the corresponding cytotoxic agents (Desa, PTX, or CPT) and CBG. In two NP pairs, an interesting difference in the cell effect was observed depending on the linker type [1,2,26]. In particular, comparison between **Np2b** and **Np2a** and between **Np3b** and **Np3a** highlight the significant increase in antiproliferative activity in NPs containing the 4,4′-dithiodibutyric acid (**Np2b** and **Np3b**) instead of the sebacic acid (**Np2a** and **Np3a**) as linkers.

In detail, **Np2b** was about 8 to 18 times more effective in inducing cell death with respect to **Np2a**, and the higher cytotoxic ability exerted by the NPs containing the 4,4′-dithiodibutyric acid with respect to those with sebacic acid was confirmed on all cell lines taken into consideration. Similarly, in accordance with the results observed for PTX, the corresponding NPs, **Np2b** and **Np2a,** exerted a similar effect on all cell types taken into consideration.

The role played by the linker on cytotoxicity is even more pronounced when considering the NP pairs containing CPT, i.e., **Np3a** and **Np3b**. Indeed, the GI_50_ values indicate a difference up to about 80 times in the LN229 and MSTO-211H cell lines, with **Np3b** always more effective than **Np2a**. In particular, in both cell lines, the presence of the disulphide bond allowed submicromolar GI_50_ values to be obtained, with a more pronounced effect on MSTO-211H where a GI_50_ of 0.050 µM was observed. This latter value appears to be of particular interest when compared with that obtained in Met-5A, i.e., 0.27 µM. In fact, this difference suggests a selectivity of **Np3b** toward mesothelioma with respect to mesothelial cells, i.e., toward cancer instead of healthy cells, thus highlighting the benefit of a GSH-sensitive linker containing a disulfide bond.

**Np1b** induced a similar cytotoxic effect on all cells taken into consideration, without any specificity toward tumor types or healthy cells. A similar behavior was observed also for **Np1a**, indicating that in these NPs, the role played by the two linkers within the cells is comparable.

**Figure 3:**
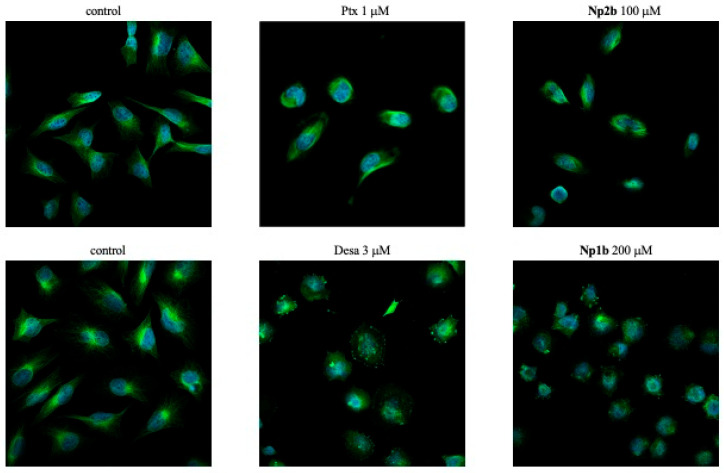
Fluorescent confocal microscope images of untreated LN229 cells (control) and those treated for 4 h with PTX, **Np2b**, Desa, and **Np1b**, at the indicated concentrations (λ_ex_/λ_em_ = 353/465 for DAPI, 493/517 for beta-tubulin). Fluorescence intensity and statistical analysis were performed, and the results are shown in Figure S2.

Taking into account that glioblastoma LN229 cells demonstrated the highest sensitivity toward CGB, and because microtubules can represent an interesting target for the treatment of glial tumors [17], we focused our attention on the mechanism of action of the most interesting NPs by analyzing the microtubule status in LN229 cells through confocal microscopy experiments. Indeed, it is well known that PTX and Desa suppress microtubule dynamics by inducing stabilizing and destabilizing effects, respectively. The micrographs in Figure 3 show untreated LN229 cells (control) and those treated with the reference agents, PTX and Desa, and the corresponding most effective NPs, **Np2b** and **Np1b,** respectively, containing 4,4′-dithiodibutyric acid as the linker.

In the cytoplasm of untreated cells (control), it was possible to observe long linear filaments, mainly oriented from the central part toward the cell’s edge. Taxol and derivatives, like PTX, act as microtubule-stabilizing agents. In particular, PTX stabilizes microtubules by suppressing dynamic changes and interfering with the assembly of the mitotic spindle, leading to failure in chromosome segregation. The most peculiar effect of such a process is the formation of microtubule bundles in interphase cells and spindle asters during mitosis. The results in Figure 3 confirm, for PTX, a polymerizing effect, and for cells treated with **Np2b,** a disturbed microtubule network comparable to that observed in the presence of PTX. Such data were confirmed by analyzing the morphological changes following incubation with the test agent, in terms of cell circularity of the control and treated cells, and through statistical analysis (Figure S2).

Treatment with 3 µM Desa for 4 h revealed a significantly dispersed signal throughout the cytoplasm due to a low number of filaments that were significantly shorter than those in the control condition, a feature consistent with the well-established depolymerizing ability of thiocolchicine. Similar changes in the filament length and arrangement, with a disorganized microtubule network without clearly defined microtubule organizer centers, were observed in the cells incubated with 200 µM **Np1b**. The correspondence between Desa and **Np1b** was further validated by analyzing the cell circularity and through statistical analysis (Figure S2).

**Figure 4:**
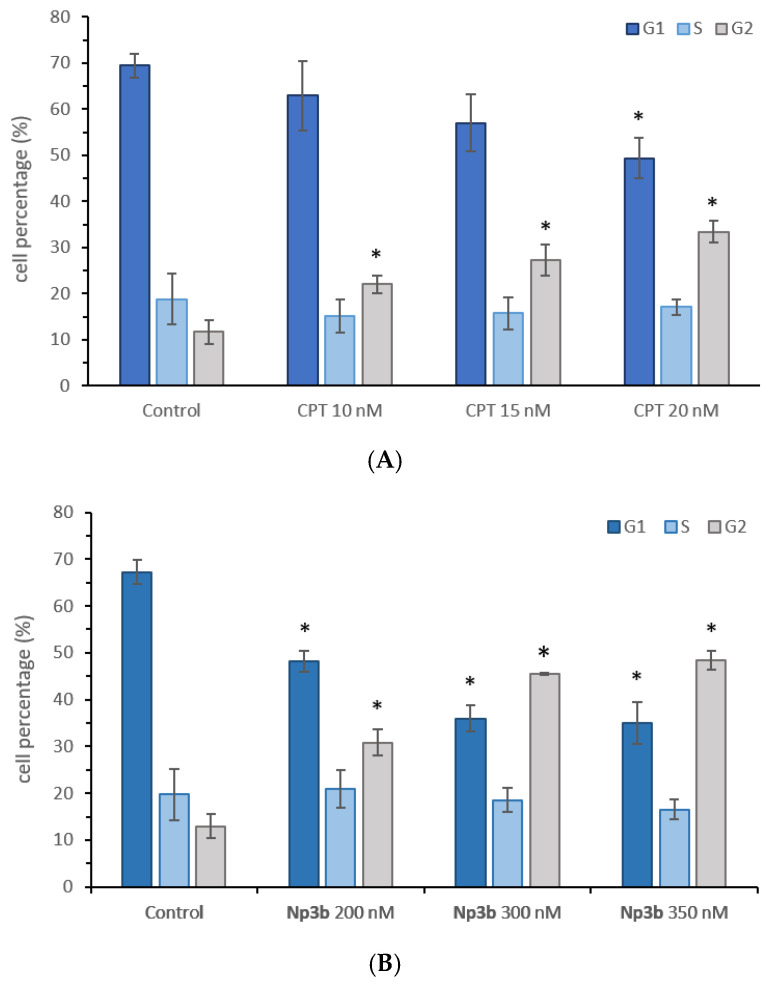
Cell cycle analysis of untreated LN229 cells (control) and treated for 48h with CPT (**A**) or **Np3b** (**B**) at the indicated concentrations. The mean values ± SD of five experiments in duplicate are reported. The statistical analysis was performed by Student’s *t*-test; *p* ≤ 0.05 (*) indicates significant difference from the corresponding control sample.

Concerning CPT and its most effective related **Np3b**, considering that the cell treatment with CPT induced an increase in the G_2_/M phase, accompanied by a decrease in G_1_,^25^ we performed cell cycle analysis of the LN229 cells treated with both agents using flow cytometry (Figure 4).

LN229 cells incubated in the presence of CPT (Figure 4A) showed a concentration-dependent increase in G_2_/M (from 11.7% in the control condition to 33.4% in the presence of 20 nM CPT) and a concurrent decrease in the G_1_ phase (from 69.4% in the control to 49.4% with 20 nM CPT). Incubation with **Np3b** at concentrations chosen in such a way that the ratio between the GI_50_ values (GI_50_Np3b/GI_50_CPT~20) was maintained induced a comparable behavior, as observable in Figure 4B, demonstrating the ability of the NPs to maintain the effect of CPT on the cell cycle.

## 4. Conclusions

In this study, we have demonstrated the remarkable potential of cannabigerol (CBG) as a useful agent for the development of multifunctional nanomaterials. Through conjugation with tubulin-targeting drugs and the incorporation of versatile linkers, we achieved the formation of stable, self-assembling nanoparticles with optimal hydrodynamic diameters (<90 nm). These nanoparticles exhibited significant antiproliferative activity against human tumor cell lines, as confirmed in glioblastoma and colorectal cells. Moreover, for one of the most effective nanoparticles, **Np3b**, the maintenance of the microtubule-targeting mechanism was confirmed in glioblastoma LN229 cells. These results represent an interesting future course of action in glioblastoma therapy; however, an in vivo study is needed to validate these findings.

## Figures and Tables

**Table 1:** Hydrodynamic diameter, polydispersity index, and zeta potential of the produced nanoformulations.

NPs (C: 100 µM)	Hydrodynamic Diameter (nm)	Polydispersity Index (PI)	ζ-Potential (mV)
**1a**	66.6 ± 1.72	0.11 ±0.048	−26.76 ± 1.09
**1b**	73.5 ± 0.94	0.13 ± 0.025	−29.2 ± 1.48
**2a**	61.03 ± 1.32	0.13 ± 0.054	−26.33 ± 1.35
**2b**	88.3 ± 2.25	0.05 ± 0.012	−29.5 ± 1.47
**3a**	68.3 ± 1.17	0.11 ± 0.058	−29.53 ± 0.46
**3b**	73.5 ± 1.25	0.11 ± 0.059	−31.82 ± 1.52

**Table 2:** Cytotoxicity of NPs and reference drugs on human tumor cell lines (LN229, HT-29, and MSTO-211H) and non-tumorigenic cells (MeT-5A).

Compound	GI_50_ (µM) ^a^			
	LN229	HT-29	MSTO-211H	MeT-5A
Desa	0.021 ± 0.001	0.015 ± 0.002	0.012 ± 0.001	0.014 ± 0.004
PTX	0.0042 ± 0.0007	0.0028 ± 0.0002	0.0033 ± 0.0006	0.0044 ± 0.0001
CPT	0.0079 ± 0.0001	0.0079 ± 0.0008	0.0033 ± 0.0010	0.0078 ± 0.0002
CBG	5.9 ± 1.3	9.7 ± 0.2	16 ± 2	16 ± 2
**Np1a**	1.0 ± 0.2	0.80 ± 0.18	1.0 ± 0.2	0.88 ±0.05
**Np1b**	1.6 ± 0.6	1.0 ± 0.1	1.7 ± 0.1	1.6 ±0.1
**Np2a**	3.7 ± 0.6	1.9 ± 0.5	6.2 ± 1.7	3.4 ± 0.5
**Np2b**	0.20 ± 0.04	0.16 ± 0.03	0.33 ± 0.11	0.40 ± 0.07
**Np3a**	13 ± 1	2.7 ± 0.2	4.2 ± 0.8	5.2 ± 0.5
**Np3b**	0.16 ± 0.01	0.21± 0.01	0.050 ± 0.003	0.27 ± 0.02

Notes: ^a^ The GI_50_ value is the concentration of the test agent able to induce a 50% reduction in the cell number with respect to the control culture. The values are the mean ± SD of at least three experiments in duplicate. The GI_50_ values were obtained by non-linear regression analysis of Nps vs. normalized response, using GraphPad Prism software version 9.0 (see Figure S1 for the most representative compounds).

## Data Availability

The datasets used and analyzed during the current study are available from the corresponding author upon reasonable request.

## Supplementary Materials

The following supporting information can be downloaded at https://www.mdpi.com/article/10.3390/pharmaceutics17050636/s1 and includes: Spectral and Characterization Data; ^1^H and ^13^C NMR Spectra; Figure S1: Cell viability of LN229 vs most representative compounds; Figure S2: Circularity Analysis performed using the built-in Analyze Particles ImageJ plugin; Figure S3: Size distribution graphs of Np3a and Np3b.
