# Supplementary material for: Self-Assembled Cannabigerol-Based Nanoparticles: Design, Synthesis, and Antiproliferative Activity
Source: Pharmaceutics. 2025 May 11;17(5):636. doi: 10.3390/pharmaceutics17050636 (PMC12114796; doi:10.3390/pharmaceutics17050636)
Supplement: Supplementary file 1 [file pharmaceutics-17-00636-s001.zip › pharmaceutics-3555031-supplementary.pdf]

Supplementary materials for:

Self-Assembled Cannabigerol-Based Nanoparticles: Design, Synthesis, and Antiproliferative Activity

Arianna Amenta [1], Giulia Nordio [2], Francesco Piazzola [2], Maria Luisa Di Paolo [3], Fabio Milani [1], Martina Giacomini [1], Andrea Citarella [1,*], Umberto Ciriello [4], Giuseppe Paladino [4], Sara Pellegrino [5], Federica Silvestri [6], Valerio Fasano [1], Lisa Dalla Via [2,*] and Daniele Passarella [1]

1 Department of Chemistry, University of Milan, Via Golgi 19, 20133 Milano, Italy; arianna.amenta@unimi.it (A.A.); fabio.milani4@studenti.unimi.it (F.M.); martina.giacomini@studenti.unimi.it (M.G.); valerio.fasano@unimi.it (V.F.); daniele.passarella@unimi.it (D.P.)
2 Department of Pharmaceutical and Pharmacological Sciences, University of Padova, via F. Marzolo 5, 35131 Padova, Italy; giulia.nordio.1@studenti.unipd.it (G.N.); francesco.piazzola@unipd.it (F.P.)
3 Department of Molecular Medicine, University of Padova, via G. Colombo 3, 35131 Padova, Italy;
marialuisa.dipaolo@unipd.it
4 LINNEA SA, 6595 Riazzino, Switzerland; uciriello@linnea.ch (U.C.); gpaladino@linnea.ch (G.P.)
5 Department of Pharmaceutical Science, University of Milan, Via Golgi 19, 20133 Milano, Italy; sara.pellegrino@unimi.it
6 Department of Industrial Engineering, University of Padova, Via Marzolo 9, 35131 Padova, Italy; federica.silvestri.3@phd.unipd.it
* Correspondence: andrea.citarella@unimi.it (A.C.); lisa.dallavia@unipd.it (L.D.V.)

Contents	Pages
**Spectral and Characterization Data**	S1 – S8
**^{1}H and ^{13}C NMR Spectra**	S9 – S14
**Figure S1.** Cell viability of LN229 vs most representative compounds.	S15
**Figure S2.** Circularity Analysis performed using the built-in Analyze Particles ImageJ plugin	S16
**Figure S3.** Size distribution graphs of Np3a and Np3b	S17

Spectral and characterization data

10-oxo-10-(2-(trimethylsilyl)ethoxy)decanoic acid (1). To a stirred solution of sebacic acid (1.00 g, 4.99 mmol) in dry CH_2Cl_2 (25 mL, 0.1 M) and dry pyridine (2.5 mL), EDCl (0.552 g, 2.89 mmol) and DMAP (0.795 g, 6.51 mmol) were added under nitrogen atmosphere at rt. After 15 minutes, 2-(trimethylsilyl)ethanol was added (0.31 mL, 2.17 mmol). The reaction was left stirring at rt overnight. After reaction completion (TLC monitoring in 7:3 *n*-hex/EtOAc + 1% HCOOH), the organic phase was washed with 1M HCl (20 mL), then the acidic aqueous phase was extracted with CH_2Cl_2 (3x10 mL). The collected organic phases were washed with brine, dried over anhydrous Na_2SO_4 and concentrated under reduced pressure. The crude was purified by flash column chromatography (silica gel, eluent mixture 8:2 *n*-hex/EtOAc + 1% HCOOH) to give 435 mg of **1** as a colorless oil, with an overall yield of 66%. ^{1}H NMR (400 MHz, $CDCl_3$): δ 4.20 – 4.11 (m, 2H, CH_2), 2.34 (t, J = 7.5 Hz, 2H, CH_2), 2.27 (t, J = 7.5 Hz, 2H, CH_2), 1.61 (h, J = 7.4 Hz, 4H, CH_2), 1.31 (d, J = 7.4 Hz, 8H, CH_2), 1.03 – 0.92 (m, 2H, CH_2), 0.04 (s, 9H, CH_3). ^{13}C NMR (100 MHz, $CDCl_3$): δ 177.93, 173.60, 62.58, 34.32, 34.22, 29.46, 29.48, 29.30, 29.10, 25.12, 25.07, 17.05, -1.53.

4-((4-oxo-4-(2-(trimethylsilyl)ethoxy)butyl)disulfaneyl)butanoic acid (2). To a solution of 4,4'-dithiodibutyrric acid (1 g, 4.19 mmol) in CH_2Cl_2 (25 mL, 0.1 M) and pyridine (2.5 mL) under nitrogen atmosphere, EDCI (0.472 g, 2.46 mmol) and DMAP (74 mg, 0.615 mmol) were added. After 15 minutes, 2-(trimethylsilyl)ethanol was added (0.26 mL, 1.84 mmol). The reaction was left stirring at rt overnight. Reaction was monitored by TLC (7:3 *n*-hex/EtOAc) and purified by flash column chromatography (8:2 *n*-hex /EtOAc) to give 413 mg of **2** as a colorless oil, with an overall yield of 66% yield. ^{1}H NMR (400 MHz, $CDCl_3$): δ 4.21 – 4.11 (m, 2H, CH_2), 2.81 – 2.71 (m, 4H, CH_2), 2.50 (t, J = 7.3 Hz, 2H, CH_2), 2.42 (t, J = 7.3 Hz, 2H, CH_2), 2.11 – 1.96 (m, 4H, CH_2), 1.04 – 0.93 (m, 2H, CH_2), 0.04 (d, 7H, CH_3). ^{13}C NMR (100 MHz, $CDCl_3$): δ 176.9, 172.9, 63.2, 36.9, 36.8, 33.4, 33.3, 24.0, 23.9, 17.2, - 1.5.

(*E*)-1-(2-(3,7-dimethylocta-2,6-dien-1-yl)-3-hydroxy-5-pentylphenyl)10-(2-(trimethylsilyl)ethyl) decanedioate (3). To a stirred solution of carboxylic acid **1** (184 mg, 0.611 mmol) in dry CH_2Cl_2 (0.05 M, 10 mL), EDCl (128 mg, 0.672 mmol) and DMAP (97 mg, 0.795 mmol) were added under nitrogen atmosphere at rt. The reaction was left stirring for 15 minutes, and then cannabigerol (309.5 mg, 0.978 mmol) was added. The reaction mixture was then left stirring overnight at rt. After reaction completion (TLC monitoring in 9:1 Hex/EtOAc), the mixture was quenched with a 1M HCl aqueous solution (10 mL) and then extracted with CH_2Cl_2 (3x15 mL). The organic phase was then dried over anhydrous Na_2SO_4, filtered, and concentrated under reduced pressure. The crude was purified by flash column chromatography (silica gel, eluent mixture 100% CH_2Cl_2) to give 208 mg of **3** as a white solid, with an overall yield of 57%. ^{1}H NMR (400 MHz, $CDCl_3$): δ 6.55 (d, J = 1.6 Hz, 1H, Ar H), 6.44 (d, J = 1.7 Hz, 1H, Ar H), 5.23 – 5.15 (m, 1H, =CH), 5.07 – 5.01 (m, 1H, =CH), 4.20 – 4.12 (m, 2H, CH_2), 3.21 (d, J = 7.0 Hz, 2H, CH_2), 2.52 (dt, J = 15.8, 7.7 Hz, 4H, CH_2), 2.27 (t, J = 7.5 Hz, 2H, CH_2), 2.06 (dq, J = 12.7, 6.7 Hz, 4H, CH_2), 1.77 (d, J = 1.3 Hz, 3H, CH_3), 1.67 (d, J = 1.6 Hz, 3H, CH_3), 1.60 (d, J = 12.6 Hz, 7H, aliphatic H), 1.44 – 1.23 (m, 14H, aliphatic H), 1.03 – 0.94 (m, 2H, CH_2), 0.92 – 0.84 (m, 3H, CH_3), 0.04 (t, J = 0.5 Hz, 9H, CH_3). ^{13}C NMR (100 MHz, $CDCl_3$): δ 174.16, 172.37,

155.65, 149.28, 142.80, 138.83, 132.15, 123.90, 121.35, 116.83, 114.54, 113.95, 62.55, 39.78, 35.58, 34.65, 34.41, 31.62, 30.79, 29.25, 26.54, 25.80, 25.10, 23.49, 22.65, 17.83, 17.48, 16.33, 14.15, -1.34.

(*E*)-2-(3,7-dimethylocta-2,6-dien-1-yl)-3-hydroxy-5-pentylphenyl 4-((4-oxo-4-(2-(trimethylsilyl)ethoxy)butyl)disulfaneyl)butanoate (4). To a stirred solution of carboxylic acid **2** (66 mg, 0.196 mmol) in dry CH_2Cl_2 (0.05 M, 3.9 mL), EDCI (41 mg, 0.215 mmol) and DMAP (31 mg, 0.255 mmol) were added under nitrogen atmosphere at rt. The reaction was left stirring for 15 minutes, and then cannabigerol was added. The reaction mixture was then left stirring overnight at rt. Reaction was monitored by TLC (9:1 *n*-hex/EtOAc) and purified by flash column chromatography (9:1 *n*-hex /EtOAc) to give 55 mg of **4** as a colorless oil with an overall yield of 43%. ^{1}H NMR (400 MHz, $CDCl_3$): δ 6.56 (s, 1H, Ar H), 6.45 (s, 1H, Ar H), 5.19 (d, *J* = 7.0 Hz, 1H, =CH), 5.08 – 4.97 (m, 1H, =CH), 4.21 – 4.14 (m, 2H, CH_2), 3.21 (d, *J* = 6.8 Hz, 2H, CH_2), 2.79 (t, *J* = 7.4 Hz, 2H, CH_2), 2.76 – 2.66 (m, 4H, CH_2), 2.51 (t, *J* = 7.8 Hz, 2H, CH_2), 2.42 (d, *J* = 7.8 Hz, 2H, CH_2), 2.21 – 1.92 (m, 8H, aliphatic H), 1.78 (s, 3H, CH_3), 1.67 (s, 3H, CH_3), 1.58 (d, *J* = 10.5 Hz, 4H, aliphatic H), 1.37 – 1.22 (m, 8H, aliphatic H), 1.03 – 0.94 (m, 2H, CH_2), 0.88 (t, *J* = 7.0 Hz, 5H, CH_3), 0.04 (s, 7H, CH_3). ^{13}C NMR (100 MHz, $CHCl_3$): δ 173.21, 171.57, 155.67, 149.18, 142.89, 139.02, 132.19, 124.28, 121.24, 116.79, 114.50, 114.08, 62.87, 39.79, 37.97, 37.80, 35.58, 32.95, 32.62, 31.63, 30.80, 26.55, 25.82, 24.40, 24.29, 23.54, 22.66, 17.86, 17.50, 16.38, 14.16, -1.34.

(*E*)-10-(2-(3,7-dimethylocta-2,6-dien-1-yl)-3-hydroxy-5-pentylphenoxy)-10-oxodecanoic acid (5). To a stirred solution of **3** (137 mg, 0.228 mmol) in dry THF (0.05 M, 2 mL), TBAF (1M in THF, 2.3 mL, 2.281 mmol) was added under nitrogen atmosphere and the resulting mixture was left stirring at rt for 4 h. After 4 h (TLC monitoring in CH_2Cl_2/MeOH 9:1) the reaction was quenched with a saturated NH_4Cl aqueous solution (10 mL) and then extracted with EtOAc (3 x 15 mL). The organic phase was then dried over anhydrous Na_2SO_4, filtered and concentrated under reduced pressure. The crude was purified by flash column chromatography (silica gel, eluent mixture CH_2Cl_2/MeOH 97:3) to give 88 mg of **5** as colorless oil, with an overall yield of 77%. ^{1}H NMR (400 MHz, $CDCl_3$): δ 6.56 (d, *J* = 1.7 Hz, 1H, Ar H), 6.43 (d, *J* = 1.7 Hz, 1H, Ar H), 5.19 (tq, *J* = 7.0, 1.3 Hz, 1H, =CH), 5.04 (tqt, *J* = 7.5, 3.2, 1.6 Hz, 1H, =CH), 3.20 (d, *J* = 7.0 Hz, 2H, CH_2), 2.60 – 2.43 (m, 4H, CH_2), 2.34 (dd, *J* = 8.5, 6.5 Hz, 2H, CH_2), 2.11 – 1.98 (m, 4H, aliphatic H), 1.79 – 1.69 (m, 3H, CH_3), 1.67 – 1.60 (m, 6H, aliphatic H), 1.57 (h, *J* = 4.3 Hz, 5H, aliphatic H), 1.48 – 1.05 (m, 12H, aliphatic H), 0.90 – 0.85 (m, 3H, CH_3). ^{13}C NMR (100 MHz, $CDCl_3$): δ 178.58, 172.39, 155.66, 149.31, 142.75, 138.58, 132.11, 123.93, 121.43, 116.90, 114.46, 113.93, 68.65, 67.80, 67.58, 59.23, 39.78, 35.59, 34.40, 33.98, 31.63, 30.80, 29.25, 29.20, 29.14, 27.94, 26.56, 25.80, 25.09, 24.81, 24.29, 23.46, 22.65, 22.32, 19.93, 17.83, 16.32, 14.15, 13.83.

(*E*)-4-((4-(2-(3,7-dimethylocta-2,6-dien-1-yl)-3-hydroxy-5-pentylphenoxy)-4-oxobutyl)disulfaneyl)butanoic acid (6). To a stirred solution of **4** (55 mg, 0.086 mmol) in dry THF (0.05 M, 1.6 mL), TBAF (1M in THF, 0.8 mL, 0.80 mmol) was added under nitrogen atmosphere and the resulting mixture was left stirring at rt for 4 h. The reaction was monitored by TLC (DCM/MeOH 95:5) and after 4 h quenched with a saturated NH_4Cl aqueous solution and extracted with EtOAc. The organic phase was then

dried over anhydrous Na_2SO_4, filtered and evaporated under reduced pressure. The crude was purified by flash column chromatography (DCM/MeOH 95:5) to give 26 mg of **6** as colorless a oil with an overall yield of 60%. ^{1}H NMR (400 MHz, $CDCl_3$): δ 6.56 (s, 1H, Ar H), 6.44 (s, 1H, Ar H), 5.18 (t, *J* = 6.8 Hz, 1H, =CH), 5.04 (t, *J* = 6.8 Hz, 1H, =CH), 3.21 (d, *J* = 7.1 Hz, 2H, CH_2), 2.83 – 2.66 (m, 6H, aliphatic H), 2.51 (d, *J* = 7.0 Hz, 4H, CH_2), 2.21 – 1.99 (m, 8H, aliphatic H), 1.78 (s, 3H, CH_3), 1.67 (s, 3H, CH_3), 1.58 (s, 5H, aliphatic H), 1.36 – 1.27 (m, 4H, CH_2), 0.88 (t, *J* = 7.0 Hz, 3H, CH_3). ^{13}C NMR (100 MHz, $CHCl_3$): δ 177.44, 171.63, 155.65, 149.18, 142.88, 138.96, 132.19, 123.89, 121.24, 116.83, 114.47, 114.07, 39.78, 37.81, 37.70, 35.57, 32.61, 32.22, 31.62, 30.79, 26.54, 25.81, 24.30, 24.04, 23.52, 22.64, 17.86, 16.38, 14.14.

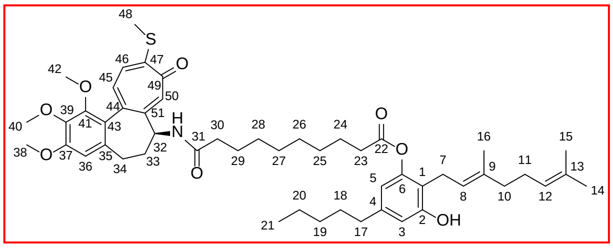

(*E*)-2-(3,7-dimethylocta-2,6-dien-1-yl)-3-hydroxy-5-pentylphenyl(S)-10-oxo-10-((1,2,3-trimethoxy-10-(methylthio)-9-oxo-5,6,7,9-tetrahydrobenzo[a]heptalen-7-yl)amino)decanoate (1a). To a stirred solution of carboxylic acid **5** (40 mg, 0.080 mmol) in dry CH_2Cl_2 (0.01 M, 5 mL), EDCl (23 mg, 0.120 mmol) and DMAP (13 mg, 0.104 mmol) were added under nitrogen atmosphere at rt. The reaction was left stirring for 30 minutes then *N*-desacetylthiocolchicine (29.9 mg, 0.080 mmol) was added. The reaction mixture was then left stirring overnight at rt. After reaction completion (TLC monitoring in CH_2Cl_2/MeOH 97:3), the solvent was removed under reduced pressure and the crude was purified by flash column chromatography (silica gel, eluent mixture CH_2Cl_2/MeOH gradient from 99.5:0.5 to 99:1) to give 4.9 mg of **1a** as a pale yellow solid, with an overall yield of 8%. ^{1}H NMR (400 MHz, $CDCl_3$): δ 7.29 (d, *J* = 10.4 Hz, 1H, **45**), 7.20 (s, 1H, **50**), 7.06 (d, *J* = 10.5 Hz, 1H, **46**), 6.55 (s, 1H, **3**), 6.52 (s, 1H, **36**), 6.42 (s, 1H, **5**), 5.23 – 5.15 (m, 1H, **8**), 5.08 – 5.00 (m, 1H, **12**), 4.63 (dt, *J* = 11.6, 6.8 Hz, 1H, **32**), 3.93 (s, 3H, **42**), 3.89 (s, 3H, **40**), 3.66 (s, 3H, **38**), 3.21 (t, *J* = 6.3 Hz, 2H, **7**), 2.56 – 2.46 (m, 6H, **17, 30, 34**), 2.43 (s, 3H, **48**), 2.37 (dt, *J* = 13.5, 7.8 Hz, 2H, **23**), 2.27 – 2.13 (m, 1H, **33'**), 2.11 – 1.91 (m, 4H, **10, 11**), 1.82 – 1.69 (m, 5H, **16, 24, 33''**), 1.69 – 1.62 (m, 3H, **15**), 1.57 (d, *J* = 6.3 Hz, 7H, **14, 18, 29**), 1.39 (s, 2H, **25**), 1.34 – 1.27 (m, 8H, **20, 26, 27, 28**), 1.38 – 1.22 (m, 2H, **19**), 0.87 (h, *J* = 3.1 Hz, 3H, **21**). ^{13}C NMR (100 MHz, $CDCl_3$): δ 134.57, 128.26, 126.42, 123.89, 121.16, 113.84, 113.84, 107.29, 61.38, 61.38, 56.11, 51.81, 39.67, 36.57, 36.57, 34.85, 34.85, 30.95, 30.95, 30.95, 29.83, 29.83, 29.83, 29.83, 25.83, 25.83, 25.83, 25.83, 23.52, 23.52, 17.82, 16.45, 14.30, 14.30. HRMS (ESI), m/z $[M + H]^+$: calculated for $C_{51}H_{70}NO_8S^+$ 856.4817; found 856.4899.

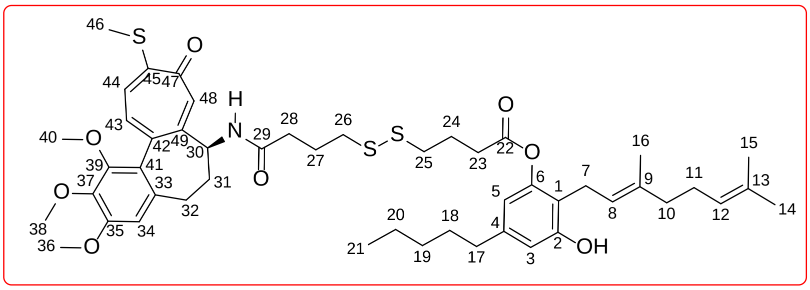

(*E*)-2-(3,7-dimethylocta-2,6-dien-1-yl)-3-hydroxy-5-pentylphenyl (*S*)-4-((4-oxo-4-((1,2,3-trimethoxy-10-(methylthio)-9-oxo-5,6,7,9-tetrahydrobenzo[a]heptalen-7-yl)amino)butyl)disulfaneyl)butanoate (1b). To a stirred solution of carboxylic acid **6** (16 mg, 0.069 mmol)

in dry CH_2Cl_2 (0.01 M, 2.9 mL), EDCI (8.35 mg, 0.044 mmol) and DMAP (4.60 mg, 0.038 mmol) were added under nitrogen atmosphere at rt. The reaction was left stirring for 30 minutes then *N*-desacetyl thiocolchicine (11.9 mg, 0.032 mmol) was added. The reaction mixture was then left stirring overnight at rt. Reaction was monitored by TLC (DCM:MeOH 97:3) and purified by flash column chromatography (DCM:MeOH 98:2) to give 9.4 mg of **1b** as a yellow solid, with an overall yield of 36 %. ^{1}H NMR (400 MHz, $CDCl_3$): δ 7.39 – 7.25 (m, 1H, **H-43**), 7.20 (d, *J* = 14.4 Hz, 1H, **H-44**), 7.13 – 7.02 (m, 1H, **H-43**), 6.76 (s, 2H, **H-48, H-34**), 6.58 – 6.55 (m, 1H, **H-5**), 6.54 – 6.48 (m, 1H, **H-3**), 6.46 – 6.39 (m, 1H, **H-30**), 5.27 – 5.14 (m, 1H, **H-8**), 5.08 – 4.97 (m, 3H, **H-12**), 3.93 (t, *J* = 2.2 Hz, 3H**, H-40**), 3.89 (d, *J* = 2.2 Hz, 3H, **H-38**), 3.72 – 3.60 (m, 3H, **H-36**), 3.21 (d, *J* = 7.0 Hz, 1H, **H-7**), 3.14 – 3.09 (m, 2H, **H-32**), 2.83 – 2.76 (m, 6H, **H-25, H-26**), 2.73 – 2.66 (m, 4H, **H-17, H-23**), 2.60 – 2.46 (m, 4H, **H-28**), 2.47 – 2.41 (m, 3H, **H-46**), 2.14 (q, *J* = 7.1 Hz, 4H, **H-31**), 2.09 – 1.90 (m, 5H, **H-10, H-11, H-24, H-27**), 1.77 (s, 2H, **H-16**), 1.72 – 1.63 (m, 10H, **H-14**), 1.61 – 1.54 (m, 9H, **H-18**, **H-15**), 1.35 – 1.23 (m, 19H, **H-19, H-20**), 0.90 – 0.85 (m, 8H, **H-21**). ^{13}C NMR (100 MHz, $CDCl_3$): δ 171.56, 171.39, 158.80, 156.28, 156.00, 154.08, 151.82, 151.06, 149.90, 142.68, 136.18, 135.36, 134.77, 131.91, 128.46, 127.30, 126.19, 124.58, 124.07, 121.69, 120.46, 118.34, 114.40, 107.88, 61.85, 56.57, 52.40, 39.98, 38.30, 37.93, 35.79, 32.90, 31.94, 31.02, 30.38, 30.17, 27.10, 26.87, 26.14, 24.47, 24.18, 23.84, 22.93, 18.18, 16.91, 15.64, 14.47. HRMS (ESI), m/z $[M + H]^+$: calculated for $C_{49}H_{65}NO_8S_3^+$ 892.2380; found 892.2301.

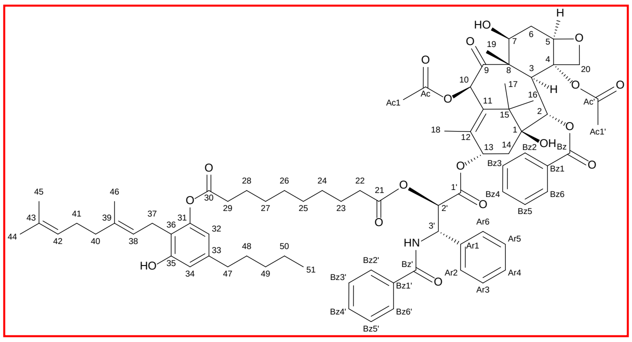

1-((1*S*,2*R*)-1-benzamido-3-(((2aR,4S,4aS,6R,9S,11S,12S,12aR,12bS)-6,12b-diacetoxy-12-(benzoyloxy)-4,11-dihydroxy-4a,8,13,13-tetramethyl-5-oxo-2a,3,4,4a,5,6,9,10,11,12,12a,12b-dodecahydro-1H-7,11-methanocyclodeca[3,4]benzo[1,2-b]oxet-9-yl)oxy)-3-oxo-1-phenylpropan-2-yl)10-(2-((*E*)-3,7-dimethylocta-2,6-dien-1-yl)-3-hydroxy-5-pentylphenyl) decanedioate (2a). To a stirred solution of carboxylic acid **5** (25 mg, 0.050 mmol) in dry CH_2Cl_2 (0.02 M, 2.5 mL) and dry DMF (0.02 M, 2.5 mL), EDCl (14 mg, 0.075 mmol) and DMAP (2.5 mg, 0.020 mmol) were added under nitrogen atmosphere at rt. The reaction was left stirring for 30 minutes then, Paclitaxel (42.7 mg, 0.050 mmol) was added. The reaction mixture was left stirring overnight at rt. After reaction completion (TLC monitoring in *n*-hex/EtOAc 1:1), the solvent was removed under reduced pressure and the crude was purified by flash column chromatography (silica gel, eluent mixture *n*-hex/EtOAc 6:4) to give 18 mg of product **2a** as a white solid, with an overall yield of 27%. ^{1}H NMR (400 MHz, $CDCl_3$): δ 8.17 – 8.10 (m, 2H, **Bz2, Bz6**), 8.00 (s, 1H, **NH**), 7.77 – 7.70 (m, 2H, **Bz2', Bz6'**), 7.65 – 7.56 (m, 1H, **Bz4**), 7.55 – 7.46 (m, 3H, **Bz4', Ar2, Ar6**), 7.45 – 7.29 (m, 7H, **Bz3, Bz5, Bz3', Bz5', Ar3, Ar4, Ar5**), 6.58 – 6.49 (m, 1H, **32**), 6.48 – 6.34 (m, 1H, **34**), 6.28 (d, *J* = 16.7 Hz, 2H, **10, 13**), 5.95 (dd, *J* = 9.2, 3.3 Hz, 1H, **3'**), 5.68 (d, *J* = 7.1 Hz, 1H, **2**), 5.51 (t, *J* = 2.4 Hz, 1H, **2'**), 5.18 (d, *J* = 7.7 Hz, 1H, **38**), 5.07 – 5.01 (m, 1H, **42**), 4.97 (dd, J = 9.7, 2.3 Hz, 1H, **5**), 4.44 (dd, *J* = 10.9, 6.6 Hz, 1H, **7**), 4.35 – 4.17 (m, 2H, **20**), 3.82 (d, *J* = 6.9 Hz, 1H, **3**), 3.21 (d, *J* = 7.1 Hz, 1H, **37**), 2.63 – 2.31 (m, 8H, **6, 22, 29, 47**), 2.45 (s, 3H, **Ac1'**), 2.22 (d, *J* = 2.6 Hz, 3H, **Ac1**), 2.20 – 1.79 (m, 6H, **14, 40, 41**), 1.94 (s, 3H, **18**), 1.78 – 1.61

(m, 13H, **19, 23, 28, 44, 46**), 1.57 (t, *J* = 4.9 Hz, 5H, **45, 48**), 1.51 – 1.24 (m, 12H, **24, 25, 26, 27, 49, 50**), 1.23 (s, 3H, **16**), 1.13 (s, 3H, **17**), 0.88 (t, *J* = 6.8 Hz, 3H, **51**). ^{13}C NMR (100 MHz, $CDCl_3$): δ 203.97, 172.87, 171.40, 169.95, 168.27, 167.20, 162.71, 142.99, 137.16, 133.82, 132.91, 132.16, 130.37, 129.33, 129.18, 128.88, 128.59, 127.23, 126.66, 84.60, 81.20, 79.32, 76.59, 75.75, 75.24, 73.95, 72.27, 71.88, 58.66, 52.96, 45.72, 43.31, 36.63, 36.03, 35.68, 34.35, 33.86, 31.58, 29.83, 29.17, 26.94, 25.80, 25.06, 24.82, 22.83, 22.64, 22.27, 20.96, 14.96, 14.14, 9.74. HRMS (ESI), m/z $[M + H]^+$: calculated for $C_{78}H_{98}NO_{18}^+$ 1336.6778; found 1336.6815.

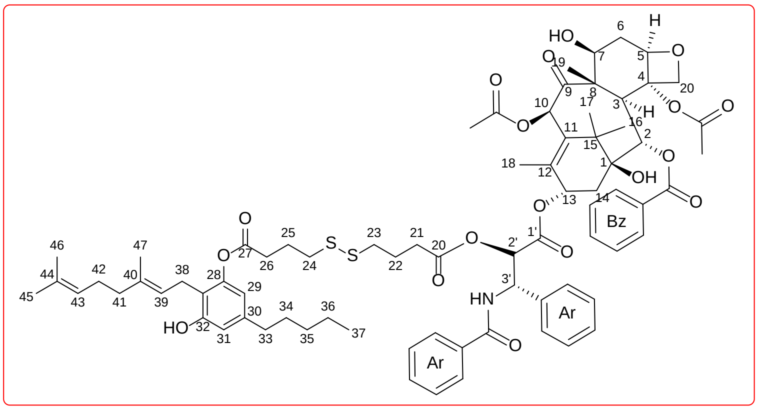

(2aR,4S,4aS,6R,9S,11S,12S,12aR,12bS)-9-(((2S,3S)-3-benzamido-2-((4-((4-(2-((E)-3,7-dimethylocta-2,6-dien-1-yl)-3-hydroxy-5-pentylphenoxy)-4-oxobutyl)disulfaneyl)butanoyl)oxy)-3-phenylpropanoyl)oxy)-12-(benzoyloxy)-4,11-dihydroxy-4a,8,13,13-tetramethyl-5-oxo-3,4,4a,5,6,9,10,11,12,12a-decahydro-1H-7,11-methanocyclodeca[3,4]benzo[1,2-b]oxete-6,12b(2aH)-diyl diacetate (2b). To a stirred solution of carboxylic acid **6** (50 mg, 0.094 mmol) in dry DMF (0.02 M, 3.9 mL), EDCI (22 mg, 0.117 mmol) and DMAP (4 mg, 0.039 mmol) were added under nitrogen atmosphere at rt. The reaction was left stirring for 20 minutes then Paclitaxel (66 mg, 0.078 mmol) was added. The reaction mixture was left stirring overnight at rt. Reaction was monitored by TLC (*n*-hex: EtOAc 1:1) and purified by flash column chromatography (*n*-hex/AcOEt 6:4) to give 37 mg of product **2b** as a white solid with an overall yield of 35%. ^{1}H NMR (400 MHz, $CDCl_3$): δ 8.14 (d, *J* = 7.9 Hz, 2H, **H-Bz**), 7.74 (d, *J* = 7.9 Hz, 2H, **H-Bz**), 7.61 (t, *J* = 7.4 Hz, 1H, **H-Bz**), 7.56 – 7.45 (m, 3H, **H-Ar**), 7.44 – 7.30 (m, 7H, **H-Ar**), 6.89 (d, *J* = 9.2 Hz, 1H, **-NH**), 6.56 (s, 1H, **H-29**), 6.43 (d, *J* = 1.6 Hz, 1H, **H-31**), 6.32 – 6.22 (m, 2H, **H-10, H-13**), 5.97 (dd, *J* = 9.2, 3.1 Hz, 1H, **H-3'**), 5.69 (d, *J* = 7.1 Hz, 1H, **H-2**), 5.51 (d, *J* = 3.1 Hz, 1H, **H-2'**), 5.18 (t, *J* = 7.0 Hz, 1H, **H-39**), 5.03 (s, 1H, **H-43**), 4.98 (d, *J* = 9.4 Hz, 1H, **H-5**), 4.45 (dd, *J* = 10.9, 6.6 Hz, 1H, **H-7**), 4.32 (d, *J* = 8.4 Hz, 1H, **H-20a**), 4.20 (d, *J* = 8.4 Hz, 1H, **H-20b**), 3.82 (d, *J* = 7.0 Hz, 1H, **H-3**), 3.20 (d, *J* = 7.0 Hz, 2H, **H-38**), 2.74 (t, *J* = 7.0 Hz, 2H, **H-24**), 2.67 (t, *J* = 7.2 Hz, 3H, **H-33, H-23**), 2.63 – 2.48 (m, 3H, **H-26, H-21),** 2.46 (s, 3H, **-OAc**), 2.38 (dd, *J* = 15.4, 9.3 Hz, 1H, **H-14a**), 2.22 (s, 3H, **-OAc**), 2.17 (s, 6H, **H-14b**), 2.14 – 1.99 (m, 8H, **H-25**, **H-22**, **H-41**, **H-42**), 1.94 (s, 3H, **H-18**), 1.76 (d, *J* = 1.4 Hz, 3H, **H-6b, H-22**), 1.68 (s, 3H, **H-19**), 1.66 (s, 4H, **H-45**), 1.60 – 1.50 (m, 5H, **H-34, H-46**), 1.33 – 1.25 (m, 4H, **H-35, H-36**), 1.23 (s, 3H, **H-16**), 1.13 (s, 3H, **H-17**), 0.88 (t, *J* = 6.8 Hz, 3H, **H-37**). ^{13}C NMR (100 MHz, $CDCl_3$): δ 203.96, 172.07, 171.55, 171.40, 169.34, 168.19, 167.22, 155.67, 149.17, 142.92, 138.88, 137.08, 133.83, 132.98, 132.20, 130.40, 129.27, 128.91, 128.64, 127.27, 126.63, 123.88, 121.26, 116.85, 114.44, 114.09, 107.29, 84.61, 81.27, 79.36, 76.59, 75.77, 75.28, 74.23, 72.29, 72.00, 60.53,

58.70, 52.87, 45.74, 43.35, 39.79, 37.65, 37.19, 35.75, 35.57, 32.59, 32.10, 31.61, 30.79, 26.97, 26.54, 25.82, 24.28, 24.03, 23.51, 22.87, 22.64, 22.30, 20.96, 17.85, 16.38, 14.98, 14.35, 14.14, 9.76. HRMS (ESI), m/z $[M + H]^+$: calculated for $C_{76}H_{94}NO_{18}S_2^+$ 1372.5907; found 1372.5986.

(*S*)-10-((4-ethyl-3,14-dioxo-3,4,12,14-tetrahydro-1*H*-pyrano[3',4':6,7]indolizino[1,2-b]quinolin-4-yl)oxy)-10-oxodecanoic acid (7). To a stirred solution of sebacic acid (122 mg, 0.603 mmol) in dry CH_2Cl_2 (0.025 M, 8 mL), EDCl (58 mg, 0.302 mmol) and DMAP (25 mg, 0.201 mmol) were added under nitrogen atmosphere at rt. The reaction was left stirring for 30 min, then Camptothecin (70 mg, 0.201 mmol) was added. The reaction mixture was then left stirring overnight at rt. After reaction completion (TLC monitoring in CH_2Cl_2/MeOH 95:5), the solvent was removed under reduced pressure and the crude was purified by flash column chromatography (silica gel, eluent mixture CH_2Cl_2/MeOH gradient from 98:2 to 97.5:2.5) to give 68 mg of **7** as a white solid, with an overall yield of 64%. ^1H NMR (400 MHz, $CDCl_3$): δ 8.41 (s, 1H, Ar H), 8.25 (d, *J* = 8.6 Hz, 1H, Ar H), 7.95 (dd, *J* = 8.3, 1.4 Hz, 1H, Ar H), 7.85 (ddd, *J* = 8.5, 6.9, 1.4 Hz, 1H, Ar H), 7.68 (ddd, *J* = 8.2, 6.9, 1.2 Hz, 1H, Ar H), 7.27 (s, 1H, Ar H), 5.68 (d, *J* = 17.2 Hz, 1H, CH_2), 5.42 (d, *J* = 17.2 Hz, 1H, CH_2), 5.31 (t, *J* = 1.4 Hz, 2H, NCH_2), 2.49 (td, *J* = 7.5, 4.8 Hz, 2H, CH_2), 2.40 – 2.31 (m, 2H, CH_2), 2.30 – 2.22 (m, 2H, CH_2), 1.64 (p, *J* = 7.1 Hz, 4H, aliphatic H), 1.53 (q, *J* = 7.2 Hz, 2H, CH_2), 1.34 (d, *J* = 7.7 Hz, 6H, aliphatic H), 0.98 (t, *J* = 7.5 Hz, 3H, CH_3). ^{13}C NMR (100 MHz, $CDCl_3$): δ 179.30, 172.90, 167.74, 157.58, 152.46, 148.92, 146.21, 131.48, 130.90, 129.62, 128.65, 128.38, 128.22, 127.64, 120.58, 96.50, 75.77, 67.24, 50.12, 34.06, 33.88, 32.00, 29.10, 29.04, 28.95, 24.77, 24.65, 7.71.

(*S*)-4-((4-((4-ethyl-3,14-dioxo-3,4,12,14-tetrahydro-1*H*-pyrano[3',4':6,7]indolizino[1,2-b]quinolin-4-yl)oxy)-4-oxobutyl)disulfaneyl)butanoic acid (8). To a stirred solution of 4,4’-dithiodibutyrric acid (86 mg, 0.360 mmol) in dry CH_2Cl_2 (0.025 M, 6 mL), EDCl (47 mg, 0.245 mmol) and DMAP (21 mg, 0.173 mmol) were added under nitrogen atmosphere at rt. The reaction was left stirring for 30 minutes then Camptothecin (50 mg, 0.144 mmol) was added. The reaction mixture was then left stirring overnight at rt. After reaction completion (TLC monitoring in CH_2Cl_2/MeOH 95:5), the solvent was removed under reduced pressure and the crude was purified by flash column chromatography (silica gel, eluent mixture CH_2Cl_2/MeOH 97:3) to give 46 mg of **8** as a pale yellow solid, with an overall yield of 56%. ^1H NMR (400 MHz, $CDCl_3$): δ 8.42 (s, 1H, Ar H), 8.27 (d, *J* = 8.5 Hz, 1H, Ar H), 7.94 (dd, *J* = 8.2, 1.4 Hz, 1H, Ar H), 7.84 (ddd, *J* = 8.5, 6.9, 1.5 Hz, 1H, Ar H), 7.67 (ddd, *J* = 8.1, 6.8, 1.2 Hz, 1H, Ar H), 7.37 (s, 1H, Ar H), 5.68 (d, *J* = 17.2 Hz, 1H, CH_2), 5.40 (d, *J* = 17.3 Hz, 1H, CH_2), 5.29 (s, 2H, CH_2), 2.81 – 2.67 (m, 5H, aliphatic H), 2.60 (dt, *J* = 16.0, 7.5 Hz, 1H, CH_2), 2.50 (td, *J* = 7.0, 4.7 Hz, 2H, CH_2), 2.28 (dq, *J* = 14.8, 7.4 Hz, 1H, CH_2), 2.22 – 2.12 (m, 1H, CH_2), 2.12 – 2.06 (m, 2H, CH_2), 2.02 (qd, *J* = 7.2, 3.3 Hz, 2H, CH_2), 0.98 (t, *J* = 7.4 Hz, 3H, CH_3). ^{13}C NMR (100 MHz, $CDCl_3$): δ 176.18, 172.21, 167.74, 157.55, 152.36, 148.62, 146.26, 145.95, 131.88, 131.12, 129.22, 128.76, 128.39, 128.34, 120.50, 96.99, 76.08, 67.19, 50.12, 38.18, 37.77, 32.54, 32.34, 31.94, 24.39, 24.08, 7.73.

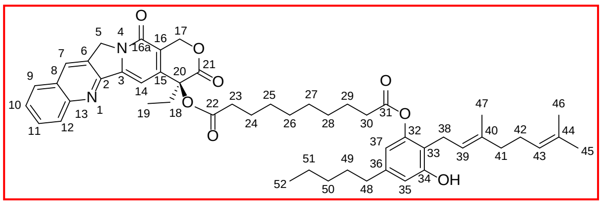

(*S*,*E*)-1-(2-(3,7-dimethylocta-2,6-dien-1-yl)-3-hydroxy-5-pentylphenyl) 10-(4-ethyl-3,14-dioxo-3,4,12,14-tetrahydro-1*H*-pyrano[3',4':6,7]indolizino[1,2-b]quinolin-4-yl) decanedioate (3a). To a stirred solution of carboxylic acid **7** (68 mg, 0.128 mmol) in dry CH_2Cl_2 (0.01 M, 10 mL), EDCl (59 mg, 0.308 mmol) and DMAP (38 mg, 0.308 mmol) were added under nitrogen atmosphere at rt. The reaction was left stirring for 30 minutes then cannabigerol (49 mg, 0.154 mmol) was added. The reaction mixture was then left stirring overnight at rt. After reaction completion (TLC monitoring in CH_2Cl_2/MeOH 95:5), the solvent was removed under reduced pressure and the crude was purified by flash column chromatography (silica gel, eluent mixture CH_2Cl_2/MeOH 98.5:1.5) and then re-purified by flash column cromatography (*n*-hex/EtOAc 4:6) to give 8 mg of **3a** as a white solid, with an overall yield of 16%. ^{1}H NMR (400 MHz, $CDCl_3$): δ 8.39 (s, 1H, **7**), 8.22 (d, *J* = 8.6 Hz, 1H, **12**), 7.97 – 7.90 (m, 1H, **9**), 7.83 (ddd, *J* = 8.5, 6.8, 1.5 Hz, 1H, **10**), 7.66 (td, *J* = 7.5, 1.2 Hz, 1H, **11**), 7.23 (s, 1H, 14), 6.55 (d, *J* = 1.7 Hz, 1H, **35**), 6.42 (d, *J* = 1.7 Hz, 1H, **37**), 5.72 – 5.61 (m, 1H, **17'**), 5.41 (d, *J* = 17.2 Hz, 1H, **17''**), 5.32 – 5.26 (m, 2H, **5**), 5.19 (t, *J* = 7.1 Hz, 1H, **39**), 5.07 – 4.99 (m, 1H, **43**), 3.20 (d, *J* = 7.0 Hz, 2H, **38**), 2.59 – 2.37 (m, 6H, **23, 30, 48**), 2.29 (dq, *J* = 14.9, 7.4 Hz, 1H, **18'**), 2.16 (s, 1H, **18''**), 2.05 (dt, *J* = 9.7, 6.7 Hz, 4H, **41, 42**), 1.78 – 1.71 (m, 3H, **47**), 1.65 (d, *J* = 7.5 Hz, 7H, **24, 29, 45**), 1.58 – 1.50 (m, 5H, **46, 49**), 1.46 – 1.26 (m, 12H, **25, 26, 27, 28, 50, 51**), 0.97 (t, *J* = 7.5 Hz, 3H, **19**), 0.88 – 0.84 (m, 3H, **52**). ^{13}C NMR (100 MHz, $CDCl_3$): δ 172.88, 172.29, 167.72, 157.55, 155.67, 152.53, 149.33, 148.97, 146.26, 146.18, 142.74, 138.59, 132.10, 131.41, 130.88, 129.68, 128.65, 128.39, 128.35, 128.21, 123.92, 121.42, 120.56, 116.92, 114.48, 113.85, 96.30, 75.80, 67.25, 50.07, 39.78, 35.57, 34.38, 33.92, 32.03, 31.61, 30.79, 29.84, 29.18, 29.05, 26.55, 25.80, 24.99, 24.76, 23.46, 22.64, 17.83, 16.33, 14.14, 7.71. HRMS (ESI), m/z $[M + H]^+$: calculated for $C_{51}H_{62}N_2NaO_8^+$ 853.4398; found 853.4271.

(*E*)-2-(3,7-dimethylocta-2,6-dien-1-yl)-3-hydroxy-5-pentylphenyl-(*S*)-4-((4-((4-ethyl-3,14-dioxo-3,4,12,14-tetrahydro-1*H*-pyrano[3',4':6,7]indolizino[1,2-b]quinolin-4-yl)oxy)-4-oxobutyl)disulfaneyl)butanoate (3b). To a stirred solution of carboxylic acid **8** (35 mg, 0.061 mmol) in dry CH_2Cl_2 (0.01 M, 5 mL), EDCl (23 mg, 0.122 mmol) and DMAP (15 mg, 0.122 mmol) were added under nitrogen atmosphere at rt. The reaction was left stirring for 30 minutes then cannabigerol (23 mg, 0.073 mmol) was added. The reaction mixture was then left stirring overnight at rt. After reaction completion (TLC monitoring in CH_2Cl_2/MeOH 95:5), the solvent was removed under reduced pressure and the crude was purified by flash column chromatography (silica gel, eluent mixture CH_2Cl_2/MeOH gradient from 99:1 to 98:2) to give 27 mg of **3b** as a white solid, with an overall yield of 51%. ^{1}H NMR (400 MHz, $CDCl_3$): δ 8.39 (s, 1H, **7**), 8.24 (d, *J* = 8.6 Hz, 1H, **12**), 7.94 (dd, *J* = 8.3, 1.4 Hz, 1H, **9**), 7.83 (ddd, *J* = 8.5, 6.9, 1.4 Hz, 1H, **10**), 7.67 (ddd, *J* = 8.2, 6.9, 1.2 Hz, 1H, **11**), 7.24 (s, 1H, **14**), 6.55 (d, *J* = 1.6 Hz, 1H, **33**), 6.43 (d, *J* = 1.6 Hz, 1H, **35**), 5.68 (d, *J* = 17.2 Hz, 1H, **17'**), 5.40 (d, *J* = 17.2 Hz, 1H, **17''**), 5.28 (dd, *J* = 2.5, 1.3 Hz, 2H, **5**), 5.23 – 5.15

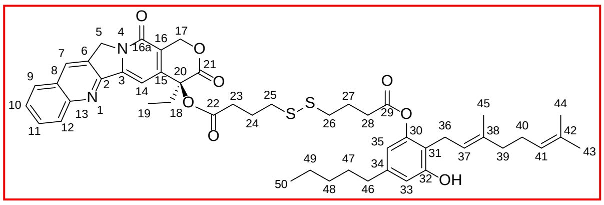

(m, 1H, **37**), 5.08 – 4.99 (m, 1H, **41**), 3.21 (d, *J* = 6.9 Hz, 2H, **36**), 2.75 (q, *J* = 7.2 Hz, 4H, **25, 26**), 2.65 (td, *J* = 7.3, 4.3 Hz, 3H, **23**), 2.61 – 2.51 (m, 1H, **28**), 2.49 – 2.44 (m, 2H, **46**), 2.32 – 1.98 (m, 8H, **18, 24, 27, 39, 40**), 1.76 (d, *J* = 1.4 Hz, 3H, **45**), 1.66 (d, *J* = 1.4 Hz, 3H, **44**), 1.58 – 1.50 (m, 5H, **43, 47**), 1.28 (tt, *J* = 8.8, 4.1 Hz, 5H, **48, 49**), 0.98 (t, *J* = 7.5 Hz, 3H, **19**), 0.90 – 0.82 (m, 3H, **50**). ^{13}C NMR (100 MHz, $CDCl_3$): δ 172.09, 171.48, 167.62, 157.51, 155.69, 152.42, 149.29, 148.87, 146.27, 146.11, 142.72, 138.40, 132.04, 131.52, 130.96, 130.42, 129.59, 128.65, 128.39, 128.26, 123.95, 121.40, 120.42, 117.03, 114.33, 113.81, 100.83, 96.31, 76.09, 67.21, 50.10, 39.78, 37.60, 37.42, 35.54, 32.63, 32.23, 31.97, 31.58, 30.76, 26.57, 25.67, 24.26, 24.11, 23.46, 22.61, 17.83, 16.36, 14.13, 7.72. HRMS (ESI), m/z $[M + H]^+$: calculated for $C_{49}H_{58}N_2NaO_8S_2^+$ 889.3527; found 889.3467.

^{1}H NMR and ^{13}C NMR spectra of final compounds

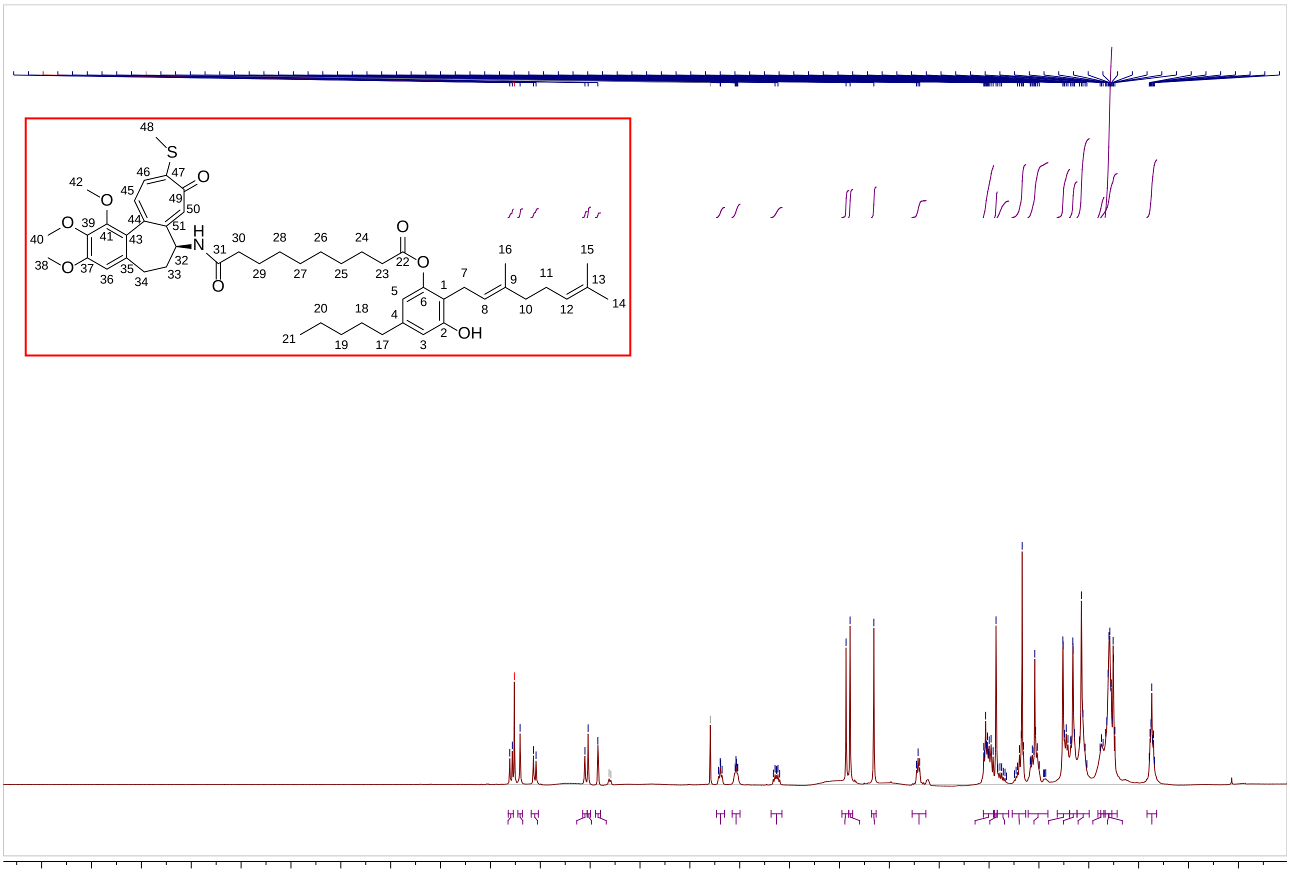

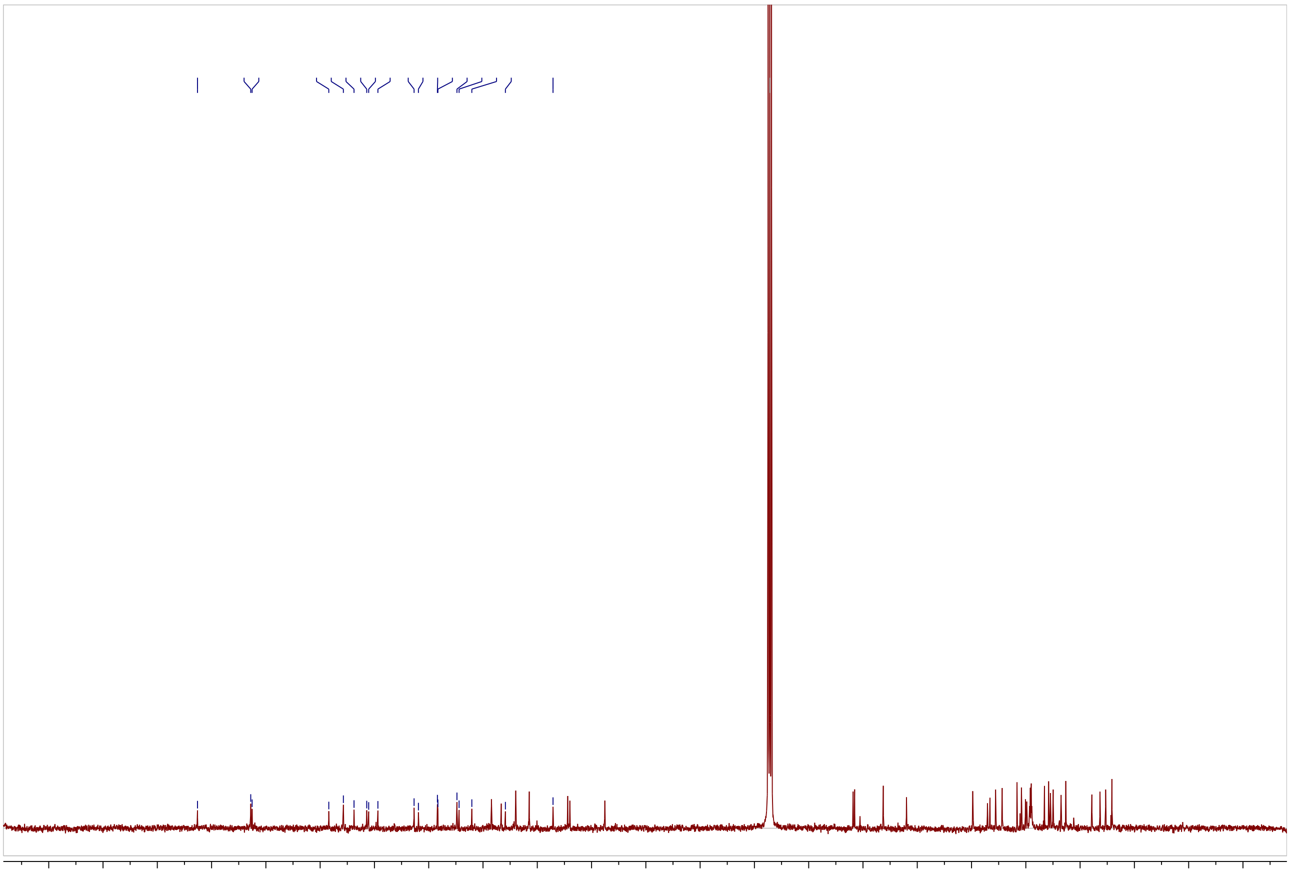

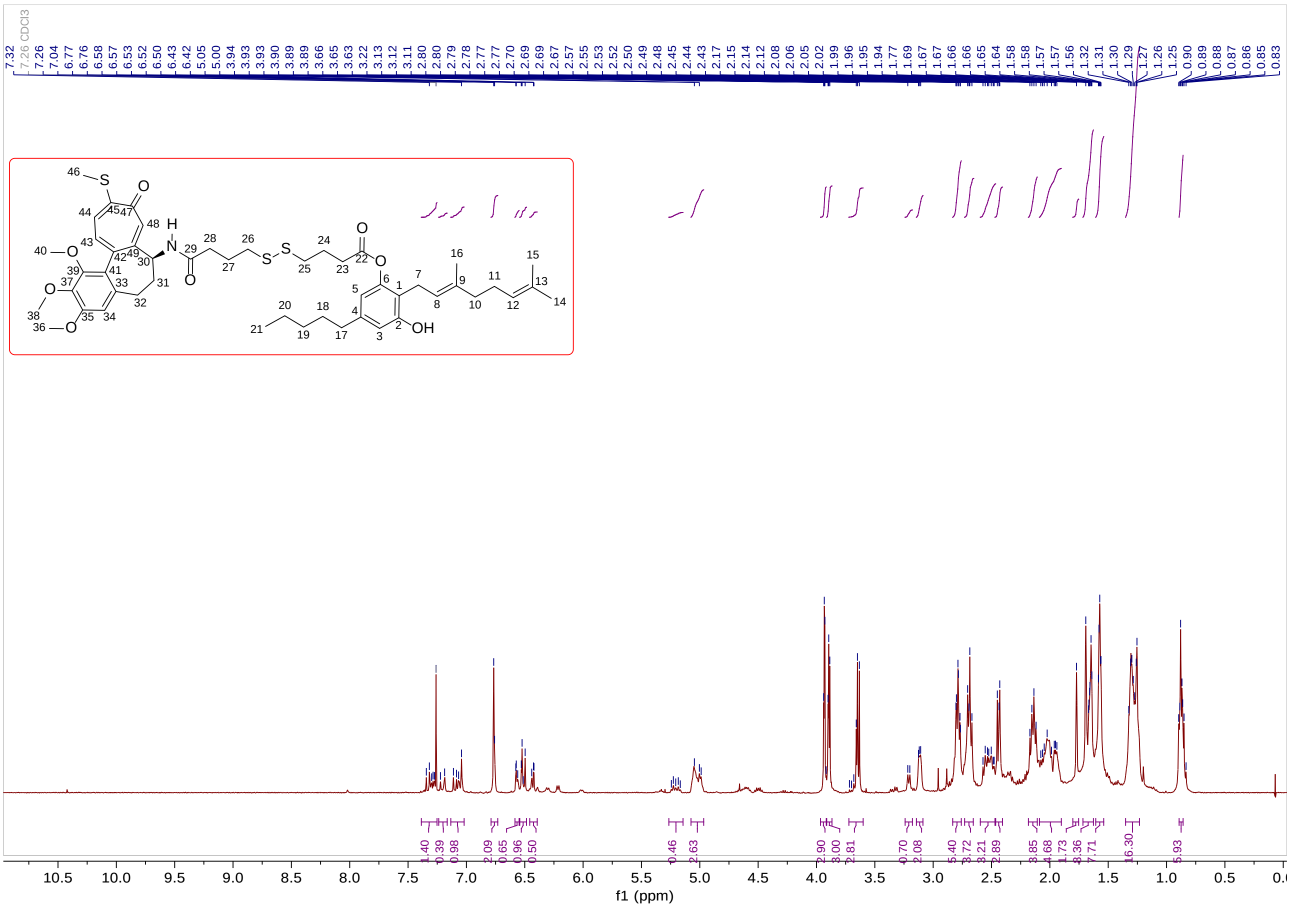
CDCl3
f1 (ppm)

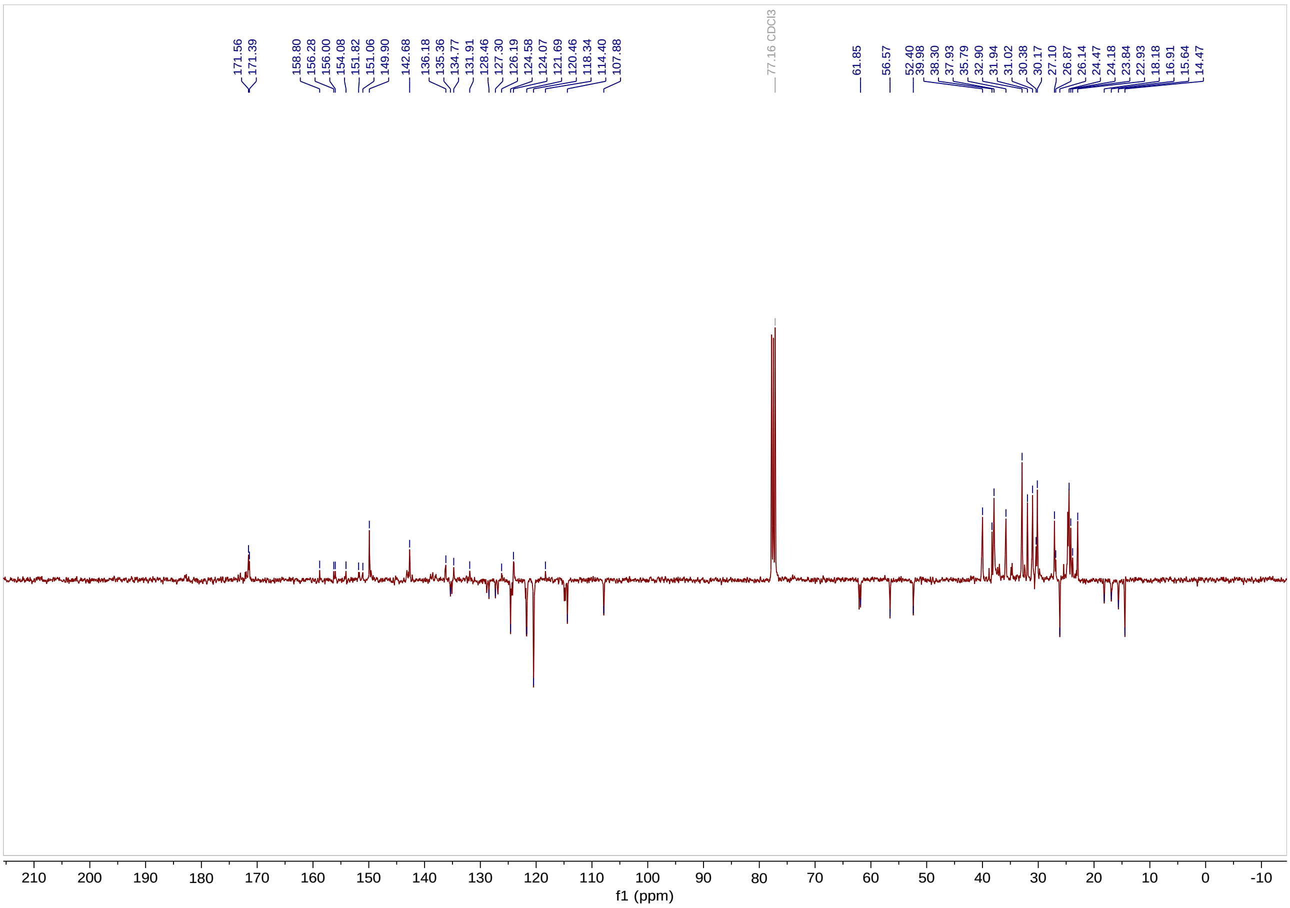
77.16 CDCl3
f1 (ppm)

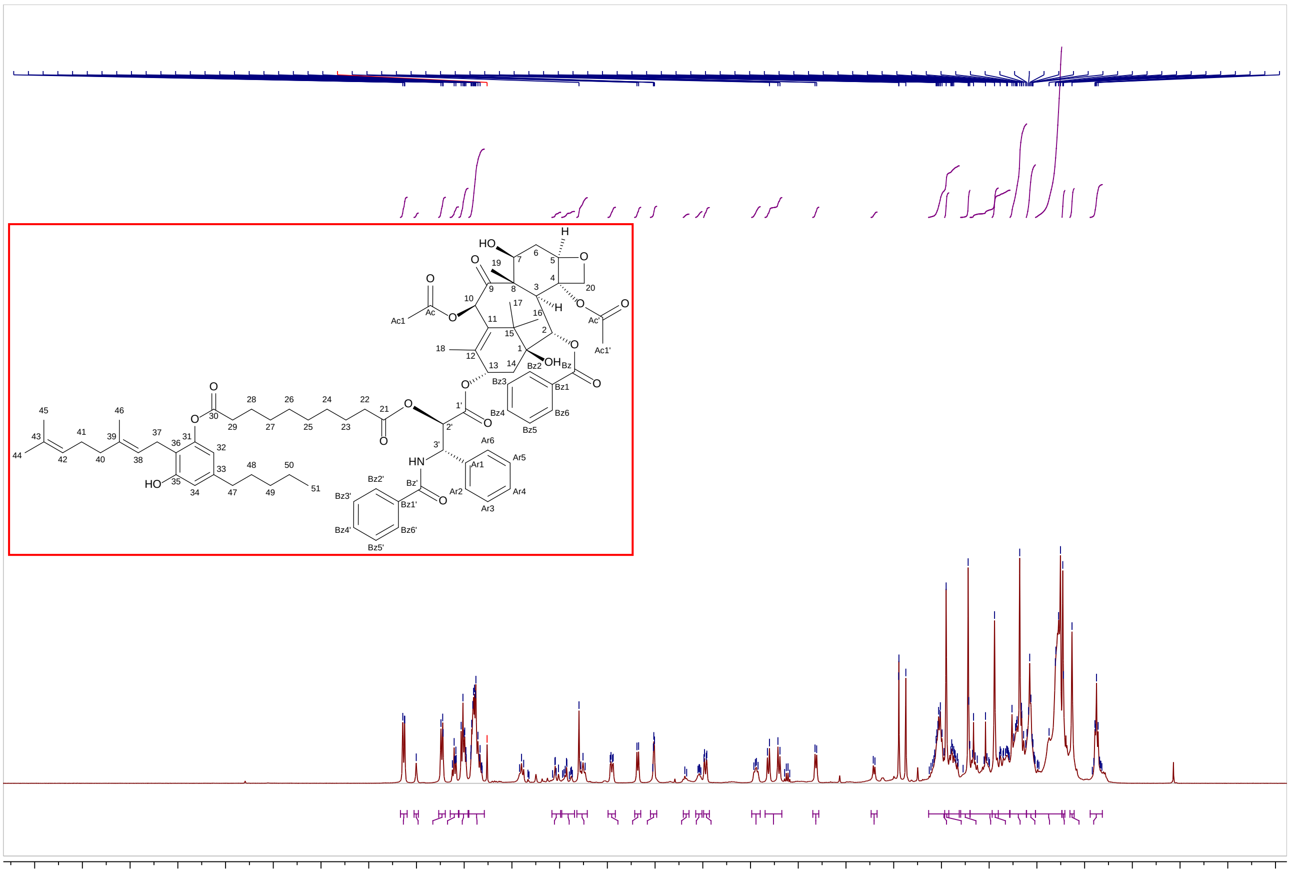
HO
HN
OH
Ac1
Ac1'

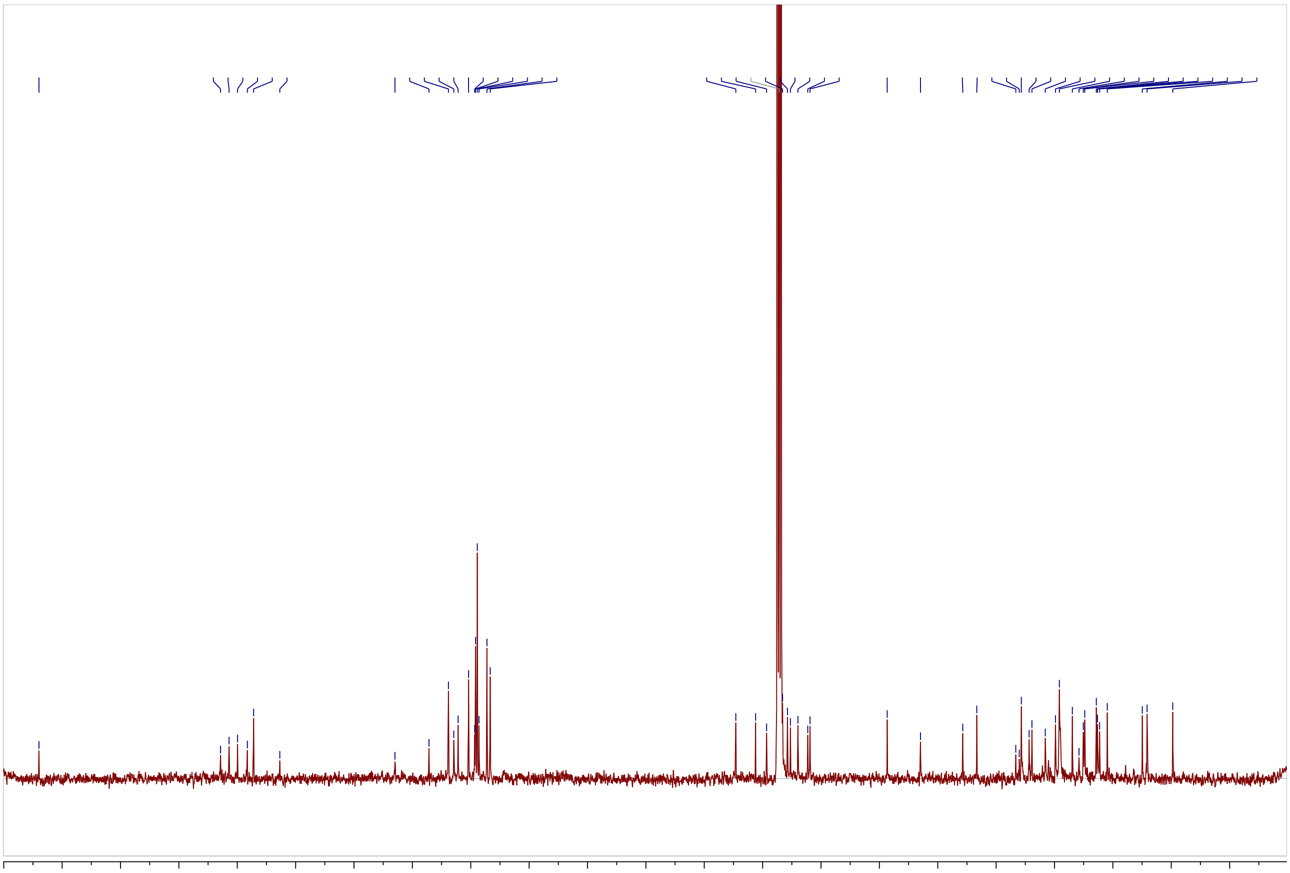

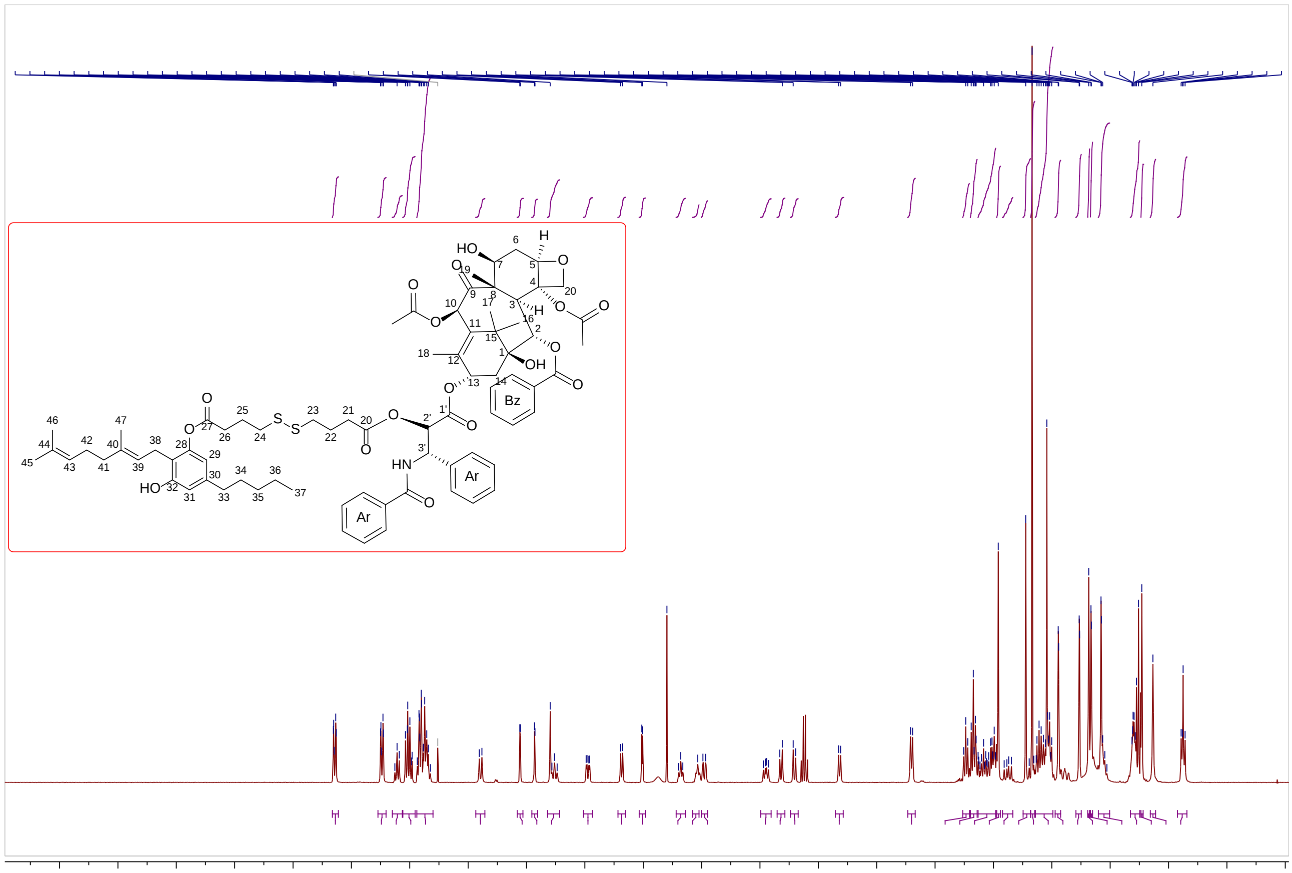
HO
Bz
HN
Ar
Ar
OH
HO

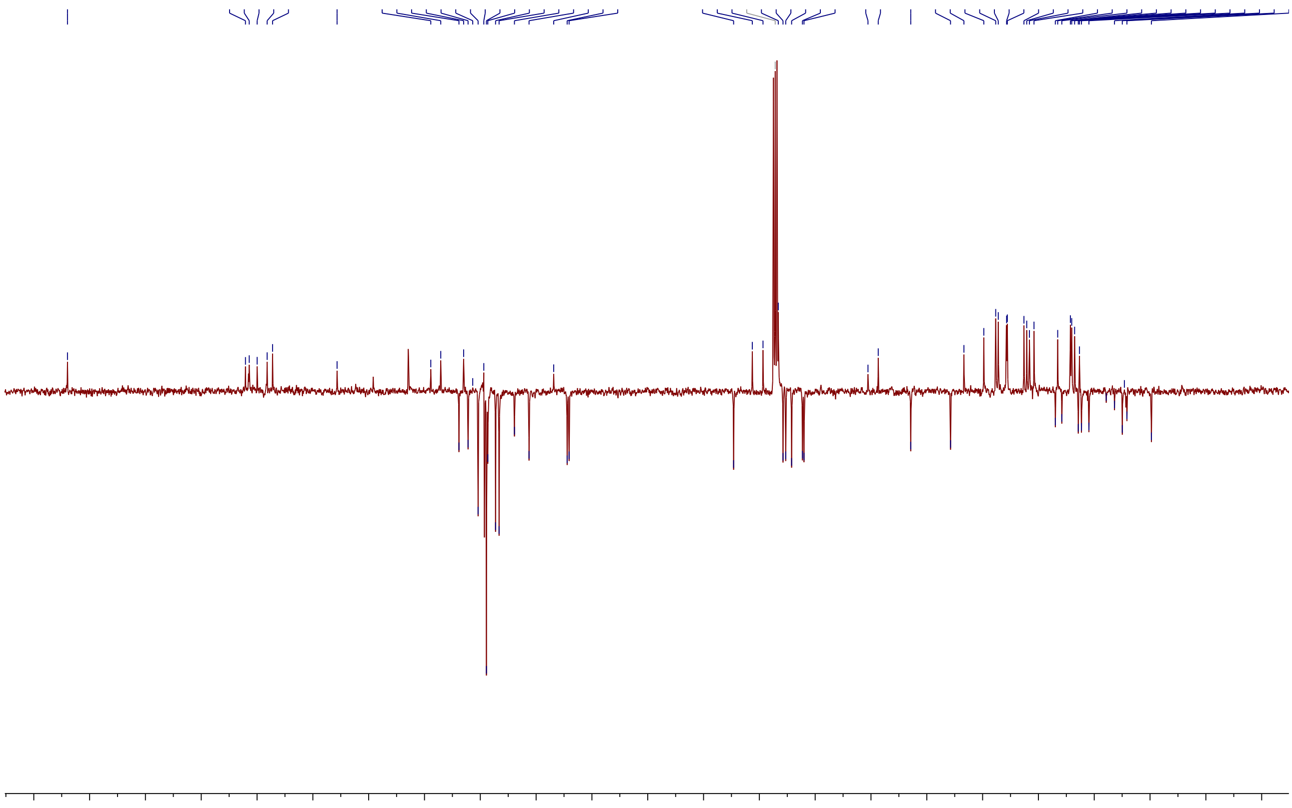

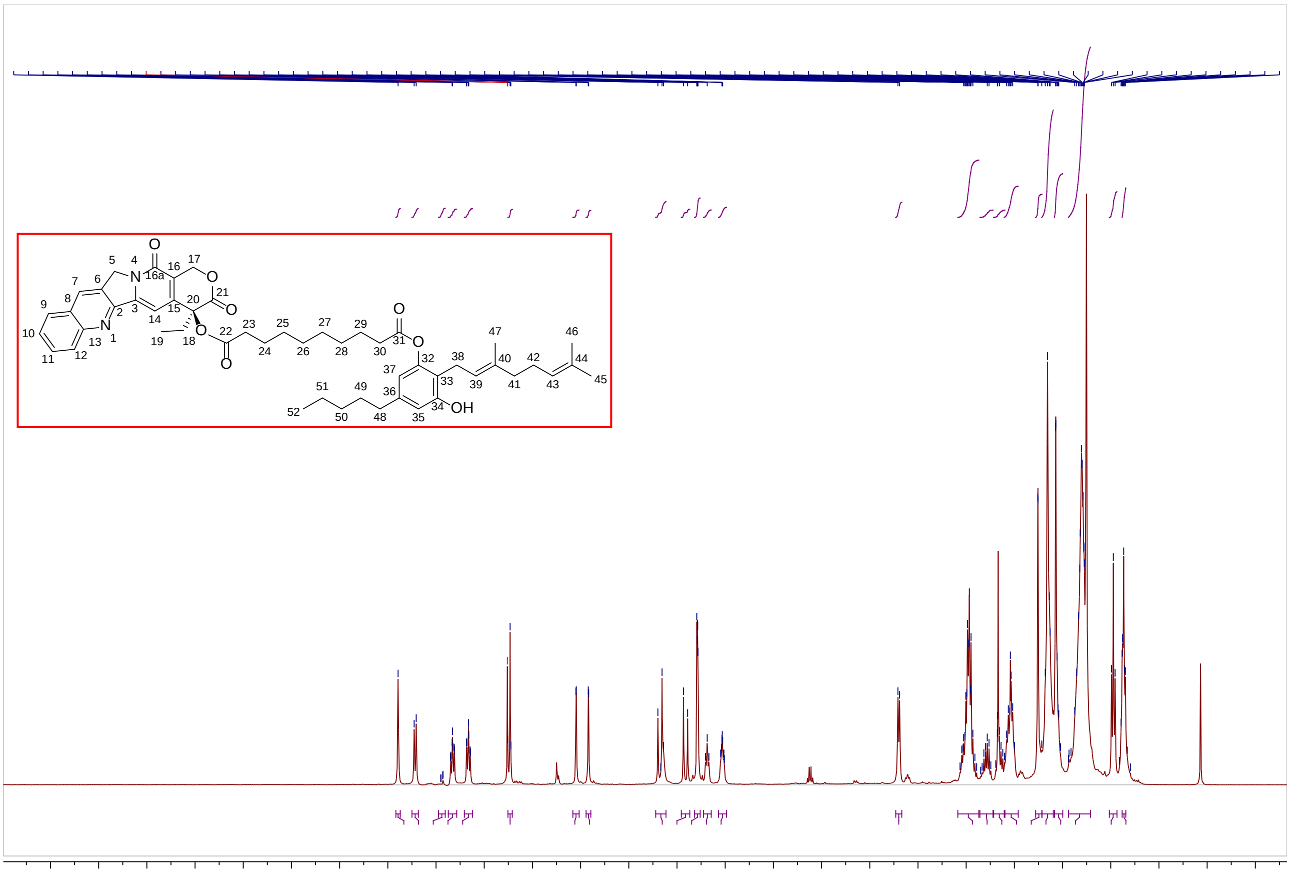

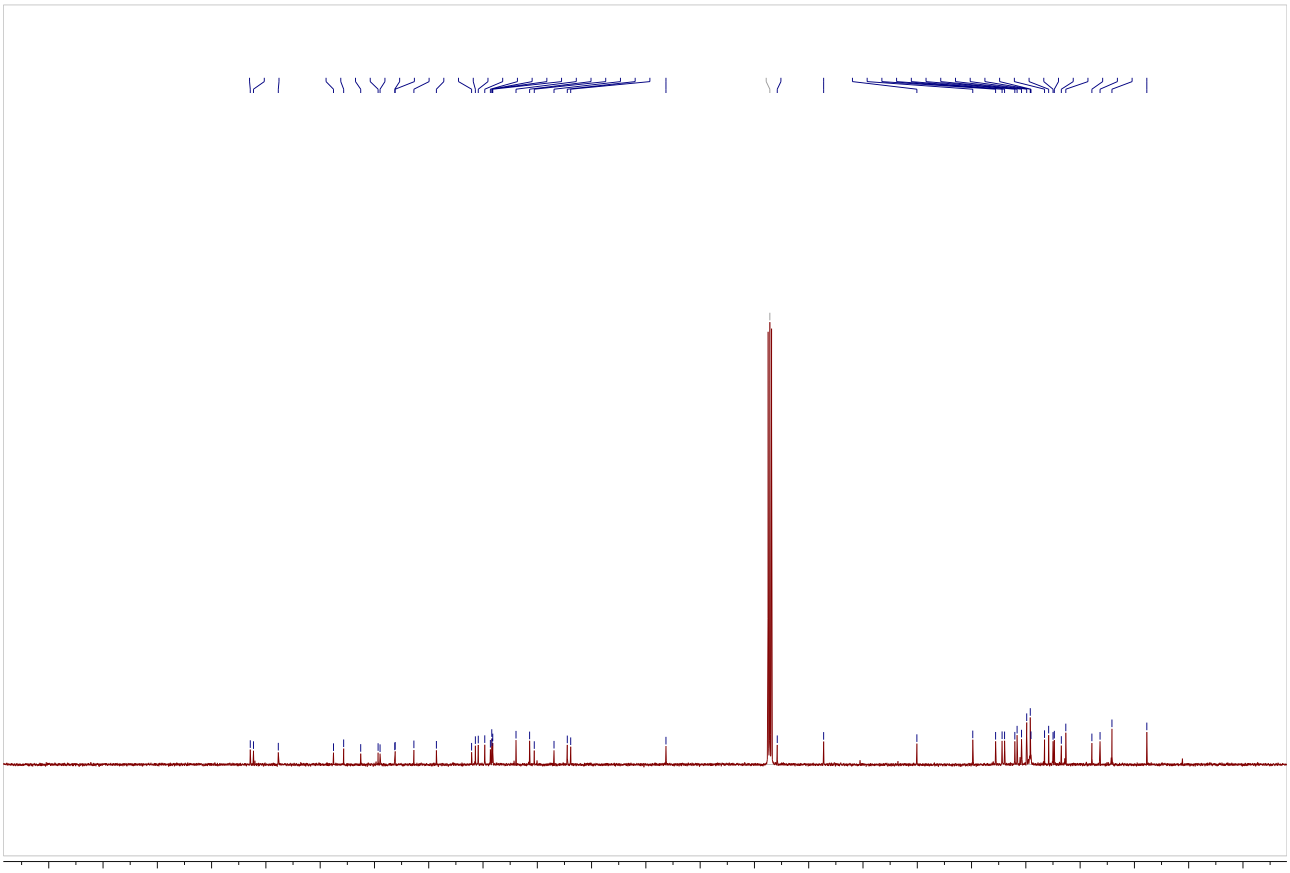

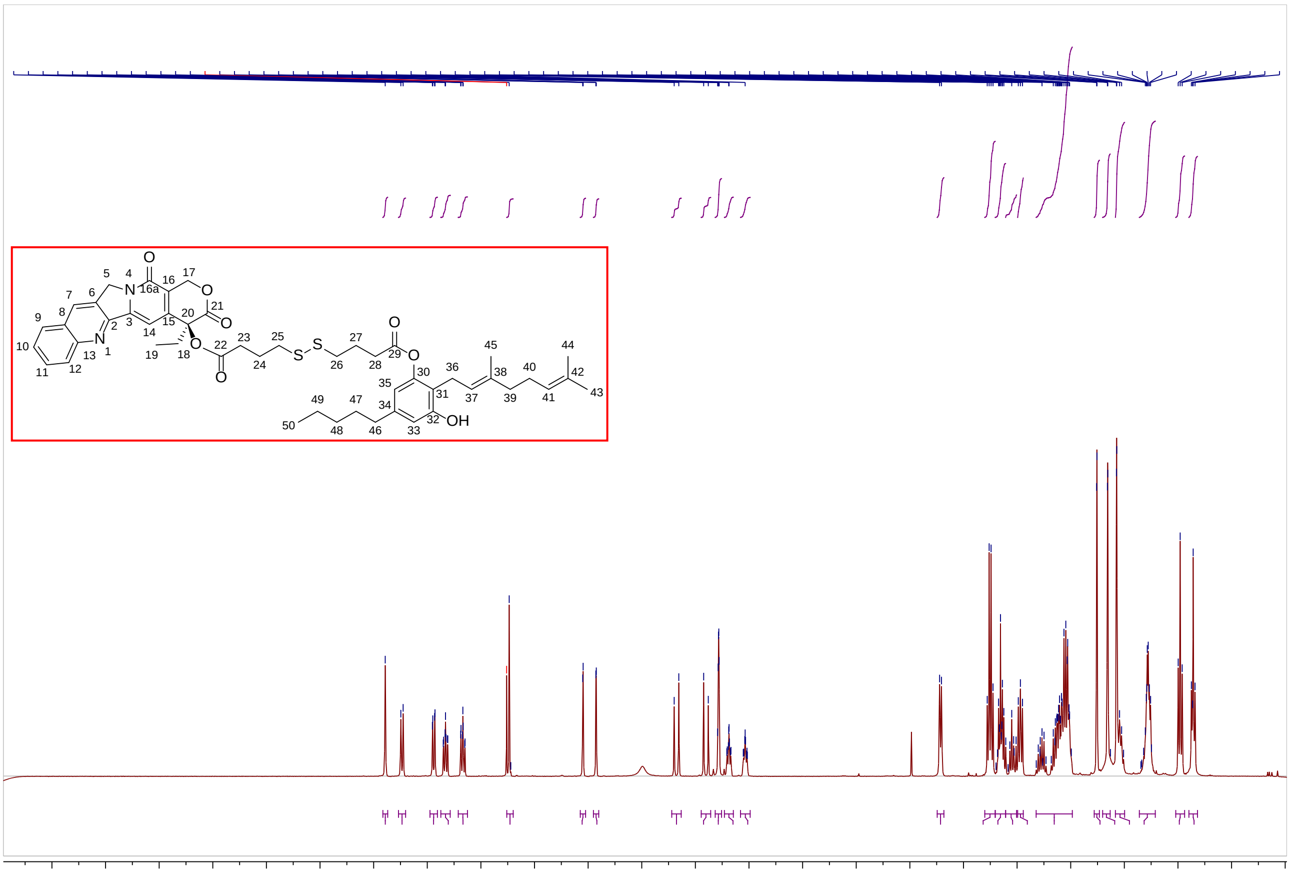

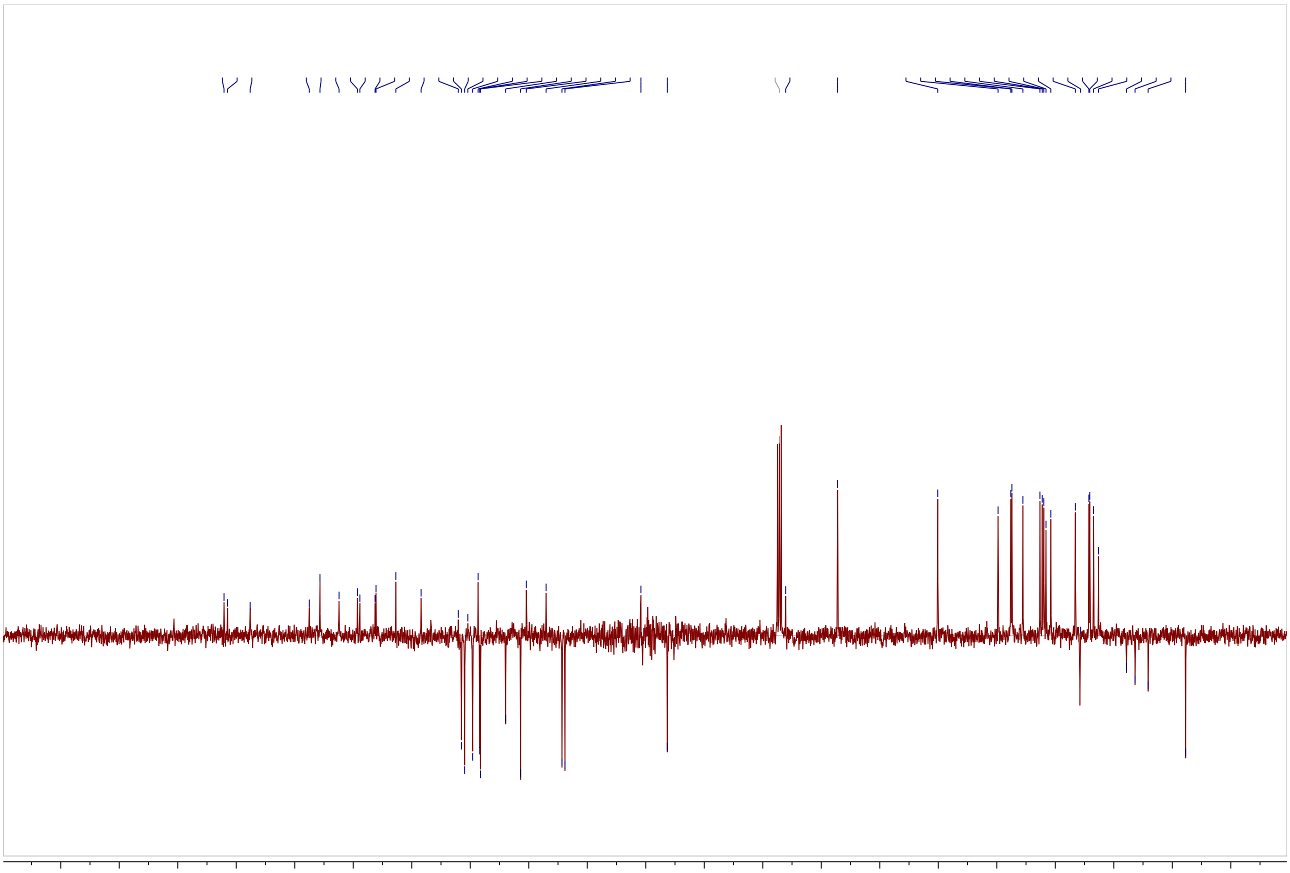

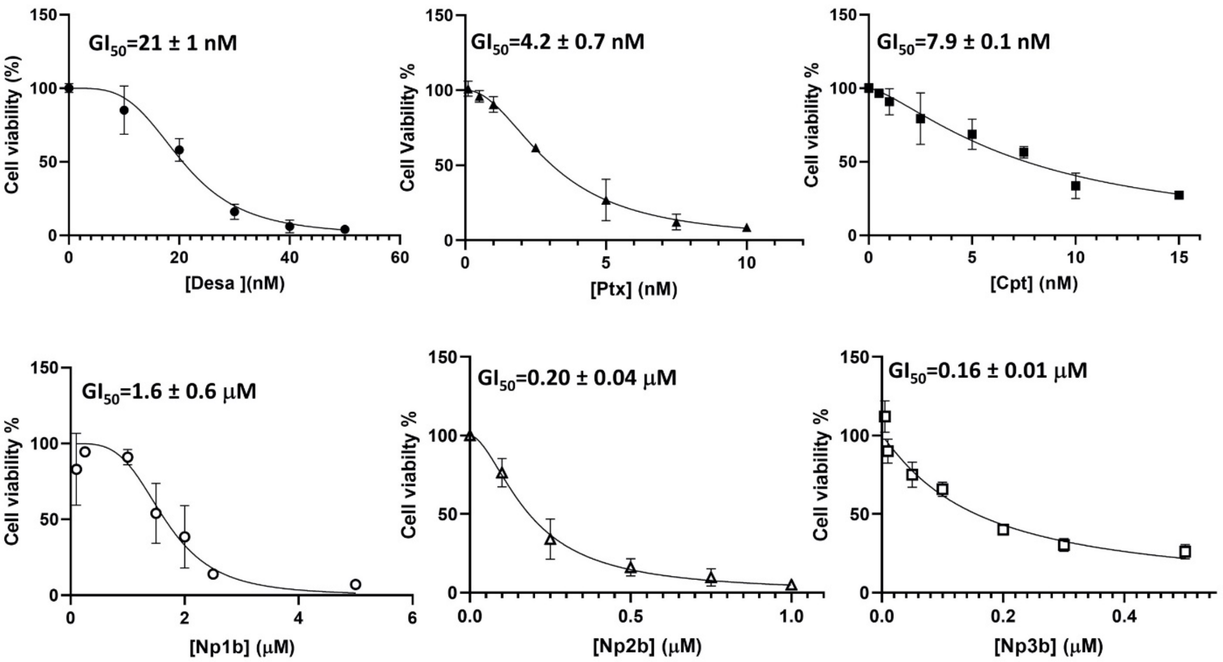

Figure S1. Cell viability of LN229 vs most representative compounds. GI_{50} values are the mean ± SD of at least three experiments in duplicate. GI_{50} values were obtained by non-linear regression analysis of [compound] vs. normalized response, using GraphPad Prism software version 9.0.

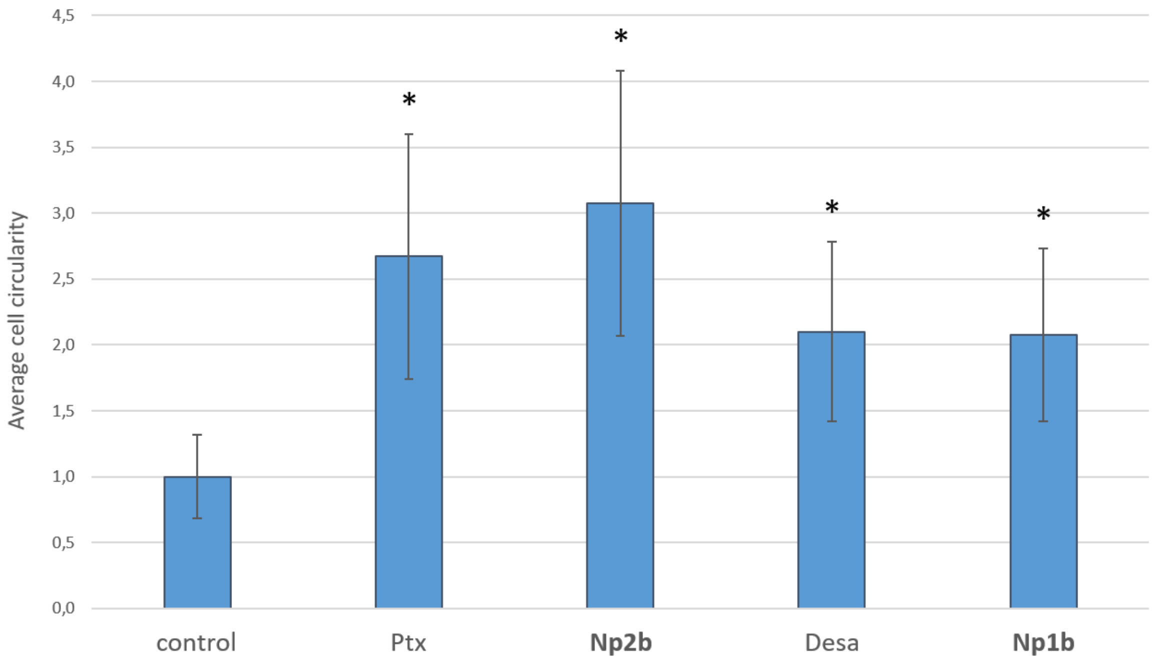

Figure S2. Circularity Analysis performed using the built-in Analyze Particles ImageJ plugin. The average circularity of the treatments (Ptx, **Np2b**, Desa, **Np1b**) were normalized to the control sample, which is set equal to 1. The statistical analysis was performed by student's T-test; $p \leq 0.05$ (*) significantly different from the control sample.

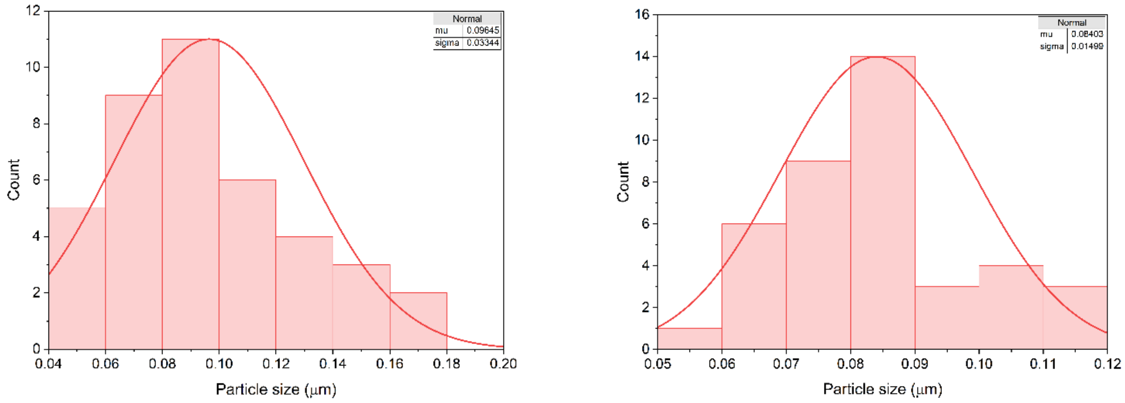

Figure S3. Size distribution graphs of **Np3a** (left panel) and **Np3b** (right panel).
